# Evolution and comparative analysis of the bat MHC-I region

**DOI:** 10.1038/srep21256

**Published:** 2016-02-15

**Authors:** Justin H. J. Ng, Mary Tachedjian, Janine Deakin, James W. Wynne, Jie Cui, Volker Haring, Ivano Broz, Honglei Chen, Katherine Belov, Lin-Fa Wang, Michelle L. Baker

**Affiliations:** 1 CSIRO Health and Biosecurity Business Unit, Australian Animal Health Laboratory, Geelong, VIC 3220, Australia; 2 Faculty of Veterinary Science, University of Sydney, NSW 2006, Australia; 3 Program in Emerging Infectious Diseases, Duke-National University of Singapore Medical School, Singapore 169857; 4 Institute for Applied Ecology, The University of Canberra, ACT 2617, Australia; 5 CSIRO, Australian Animal Health Laboratory, Geelong, VIC 3220, Australia

## Abstract

Bats are natural hosts to numerous viruses and have ancient origins, having diverged from other eutherian mammals early in evolution. These characteristics place them in an important position to provide insights into the evolution of the mammalian immune system and antiviral immunity. We describe the first detailed partial map of a bat (*Pteropus alecto*) MHC-I region with comparative analysis of the MHC-I region and genes. The bat MHC-I region is highly condensed, yet relatively conserved in organisation, and is unusual in that MHC-I genes are present within only one of the three highly conserved class I duplication blocks. We hypothesise that MHC-I genes first originated in the β duplication block, and subsequently duplicated in a step-wise manner across the MHC-I region during mammalian evolution. Furthermore, bat MHC-I genes contain unique insertions within their peptide-binding grooves potentially affecting the peptide repertoire presented to T cells, which may have implications for the ability of bats to control infection without overt disease.

In recent years, bats have been in the spotlight due to the wide recognition that they are natural reservoirs to numerous zoonotic viruses^1^,^2^,^3^,^4^. Although many bat-borne viruses are capable of causing serious disease in other susceptible hosts, they rarely result in clinical signs of disease in bats^5^,^6^,^7^,^8^,^9^,^10^. Bats also appear to carry more zoonotic viruses than other reservoir hosts, including rodents^11^. Recent analysis of bat genomes has revealed evidence of positive selection on genes involved in DNA repair and innate immunity pathways, providing evidence that the evolution of flight may have had inadvertent consequences for the innate immune system of bats^12^. Thus, co-evolution of bats with viruses, combined with the selective pressures associated with the evolution of flight, have likely played important roles in shaping their immune system.

Bats belong to the order Chiroptera and are further divided into two suborders; Yinpterochiroptera and Yangochiroptera. The Yinpterochiroptera suborder (also known as Megachiroptera) includes all megabats and three microbat families while the Yangochiroptera (also known as Microchiroptera) includes all remaining microbat families^13^,^14^. Bats are one of the most ancient extant lineages of eutherian mammals, believed to have diverged from other eutherian mammals approximately 88 million years ago (mya), with Yinpterochiroptera and Yangochiroptera having diverged from each other approximately 68 mya^12^. Bats fill an important phylogenetic gap between previously studied mammalian species, potentially bridging the 160 million year gap between marsupials and other higher order eutherians.

The major histocompatibility complex (MHC) is arguably the centre of the immune system, with genes within this region involved in both the innate and adaptive immune responses, including those responsible for the processing and presentation of foreign antigens^15^,^16^,^17^. The MHC region has now been characterised in a variety of species of mammals representing eutherians, marsupials and monotremes^18^,^19^,^20^. The MHC region is divided into Class I, II and III regions, organised along the chromosome in a Class I-III-II arrangement^15^,^17^,^19^,^21^,^22^,^23^,^24^,^25^. The MHC class I (MHC-I) region can be further divided into the extended sub-region and the classical sub-region. The extended sub-region spans from the histone H2A type 1-A (*HIST1H2AA*) gene to the myelin-oligodendrocyte glycoprotein (*MOG*) gene and contains numerous butyrophilin, histone, olfactory receptor and zinc finger loci in between^22^. The classical sub-region spans from DNA-directed RNA polymerase I subunit RPA12 (*ZNRD1*) to the MHC-I polypeptide-related sequence B (*MICB*) gene and typically contains numerous MHC-I genes^17^,^22^.

The classical MHC-I sub-region contains a set of framework genes with highly conserved presence and order amongst all mammals^20^,^26^,^27^. Amadou^26^ proposed that this framework represents an ancestral structure, and concluded that alterations of the framework could result in deleterious consequences. In eutherian mammals, MHC-I genes are located within a few permissive locations along this framework, with these places arbitrarily named the α, κ and β duplication blocks^27^. The α duplication block is demarcated by *MOG* and RING finger protein 39 (*RNF39*), the κ duplication block by tripartite motif containing 26 (*TRIM26*) and ATP-binding cassette sub-family F member 1 (*ABCF1*), and the β duplication block by transcription factor 19 (*TCF19*) and *MICB* framework genes. The MHC-I region achieved its high MHC-I gene diversity and density by allowing major perturbations, such as class I gene duplications, within these duplication blocks without disrupting the essential framework genes^27^. The framework structure also exists in the MHC-I region of sequenced representatives of lower vertebrates, marsupials and monotremes. Lower vertebrates, including teleost, *Xenopus* and chickens, have a conserved framework gene organisation but their MHC-I genes are not located within this framework; instead they are embedded within the class II region^28^,^29^,^30^,^31^. Similarly, the opossum and platypus MHC also contains a class I/II region, while in the tammar wallaby the class I genes are scattered throughout the genome^18^,^20^,^32^. The class I genes are believed to have relocated into the class I region after the divergence of marsupials and eutherians approximately 160 mya^19^,^20^,^33^.

MHC-I molecules are membrane-bound surface proteins comprising a three-domain alpha heavy chain (α1, α2 and α3), encoded within the MHC region (Chromosome 6 in human), non-covalently bound with the β_2_-microglobulin light chain (β_2_M), which is encoded outside the MHC region (Chromosome 15 in human)^34^,^35^. Class I genes can be further divided into classical (class Ia) and non-classical (class Ib) class I genes. Class Ia molecules are ubiquitously expressed, highly polymorphic and present peptide to cytotoxic T cells. Class Ib molecules are typically less polymorphic, may have tissue specific expression patterns and encode molecules with a variety of functions other than antigen presentation. Class Ia molecules are typically encoded within the MHC while class Ib molecules (such as CD1) are sometimes located outside the MHC. The hyper-variable amino-terminal α1 and α2 domains in the heavy chain of class Ia molecules form a vital peptide-binding groove (PBG), in which both ends are blocked in a closed conformation and are only large enough to accommodate a single peptide of 8–11 aa (or amino acid residues) in length^34^,^36^,^37^,^38^,^39^. The α3 domain, on the other hand, contains invariant residues critical for binding to the T cell co-receptor CD8^34^,^40^. As class Ib genes play roles other than antigen presentation, their PBG is often folded into a more narrow groove, depending on the function of the molecule^41^.

The MHC region is the most gene dense and polymorphic region of the genome and plays important roles in immunity and reproductive success, yet there is little information on the MHC of any bat species. The only previously reported bat MHC-I sequences are seven distinct but partial class I genes identified in a transcriptome dataset from *P. alecto*^42^. The diversity and polymorphism of MHC class II genes across various microbats have also been previously described in some detail^43^,^44^,^45^,^46^. However, no studies have explored the MHC-I region and repertoire in any bat species prior to our study. Here we describe the characterisation of a bat MHC-I region, using the Australian black flying fox (*P. alecto*), a megabat within the suborder Yinpterochiroptera. The content and organisation of this region was compared with other mammals, filling an important phylogenetic gap and providing insights into the duplication of class I genes within the mammalian MHC-I region. The presence of a unique insertion within the PBG of a number of the bat MHC-I molecules also provides evidence for differences in the PBG, which may reflect functions other than antigen presentation or alternatively influence the size of peptides presented to cytotoxic T cells. To our knowledge, this is the first genomic map of a bat MHC-I region and characterisation of MHC-I genes of any bat species.

## Results

### Identification of the Bat MHC-I Region from the Whole Genome

**Figure 1:**
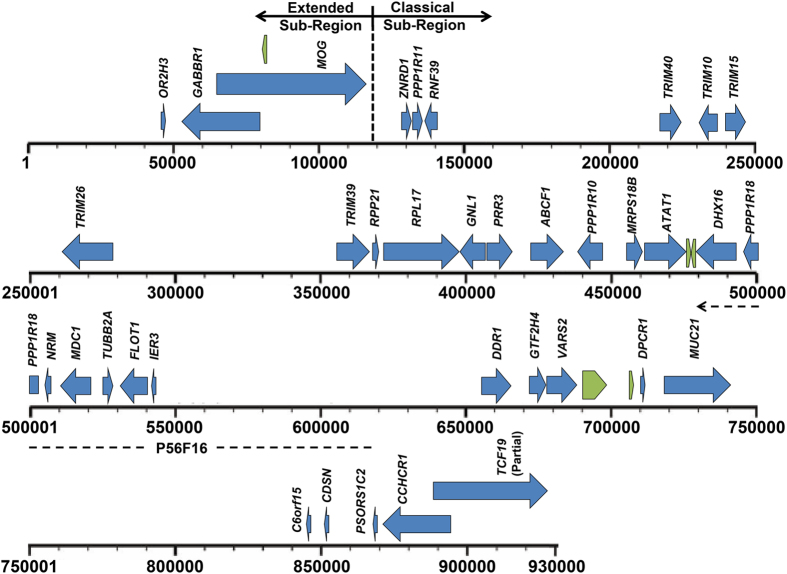
Map of the bat MHC-I region (927,264 bp) identified on scaffold555 of the *P. alecto* genome. Blue arrows represent annotated genes and green arrows represent predicted open reading frames for which there is no functional ortholog in existing databases. The black dashed line below the map corresponds to the location of BAC contig P56F16. Transcriptional orientation of genes is indicated by the direction of the arrows.

The whole genome of *P. alecto* is among the recently completed bat genomes that have been sequenced using next generation Illumina sequencing technology^12^. Using BLAST searches against the genome, one scaffold (scaffold555) was identified that contained genes corresponding to the partial mammalian classical MHC-I sub-region and was annotated and manually verified. The genomic map is illustrated in Fig. 1, with coordinates and accession numbers of the predicted genes listed in Table 1. Genes were annotated based on their similarity to orthologous genes in other species. The bat MHC-I region consists of 927,264 base pairs (bp) of contiguous sequence, spanning from flanking genes olfactory receptor 2H3 (*OR2H3*) in the extended MHC-I sub-region to *TCF19* in the classical MHC-I sub-region. Five novel open reading frames (ORFs) with no homologues were also found. Interestingly, no MHC-I genes were identified within this scaffold. Two other scaffolds, scaffold7320 (898 bp) and C18352586 (1,145 bp), containing only partial MHC-I α3 domain sequences were also identified. Due to the small size of these scaffolds and the absence of framework genes to orient them, they were not used for further analysis in this study.

To identify sequences corresponding to the conserved β duplication block, 3′ downstream of *TCF19*, manual BLAST searches of the *P. alecto* genome were performed to identify conserved framework genes, *MICB* and psoriasis susceptibility 1 candidate 3 (*PSORS1C3*) which flank the β duplication block in the human MHC-I region. *PSORS1C3* was not identified in the *P. alecto* genome. A 709bp contig (C17864126) from the *P. alecto* genome contained sequence with homology to mammalian *MICB* but contained multiple stop codons, indicating that it may represent a pseudogene. A putative *MICB* ortholog was also identified in the genome of the closely related bat, *Pteropus vampyrus* but the scaffold encoding this gene was only 8,803bp (scaffold 23172) and contained no other MHC-I or framework genes.

For comparative purposes, we also examined the genomes of other available bat species for evidence of an assembled MHC-I region. Of the publicly available bat genomes, the genome of the big brown bat (*Eptesicus fuscus*) contained a scaffold (scaffold00164) corresponding to the complete MHC-I region. *E. fuscus* is a microbat and a member of the Yangochiroptera suborder. As *P. alecto* belongs to the suborder, Yinpterochiroptera, the two bat species provide a comparison between the two suborders of bats.

### Comparative Analysis of the Bat MHC-I Region

**Figure 2:**
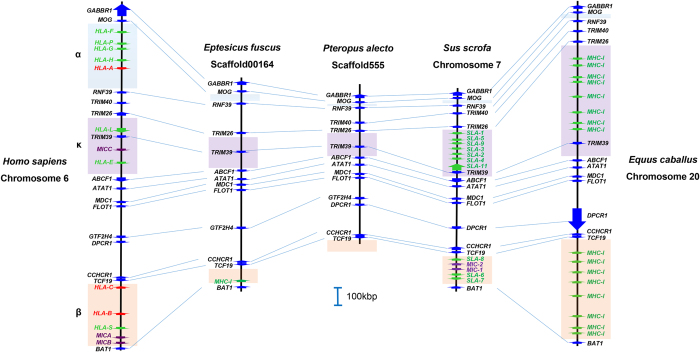
Comparative gene maps of bat MHC-I classical sub-region (centre) against corresponding regions of human and horse. Red arrows represent classical MHC-I genes, green arrows represent non-classical or undefined MHC-I genes, blue arrows represent flanking MHC-I region genes and purple arrows represent MIC genes. The areas highlighted blue, purple and orange represent the α, κ and β duplication blocks respectively. The human, horse and pig gene maps were adapted from the Ensembl annotation while the microbat gene map was adapted from NCBI annotation. Transcriptional orientation of genes is indicated by the direction of the arrows. For the MHC-I regions, not all annotated genes are shown.

The bat classical MHC-I sub-region, identified in the *P. alecto* genome was compared with the corresponding region from human, horse, pig and a microbat (*E. fuscus*). Of the species whose genome has been sequenced to date, horses are the closest living relative to bats, the two having shared a common ancestor ~88 mya^12^. The human MHC-I region was used as a reference since it is extensively annotated. The pig was included as a representative Laurasiatherian mammal because it shares the comparatively condensed MHC-I region, similar to that of *P. alecto*. The *P. alecto* gene map was constructed using the ~900 kilobases (kb) MHC-I region obtained from the bat whole genome sequence. The region from framework gene gamma-aminobutyric acid B receptor 1 (*GABBR1*) through to the end of the β duplication block of the MHC-I region of human, horse, pig and microbat was used for comparison. Resulting comparative gene maps revealed conserved framework gene content and organisation across all five species (Fig. 2). Similar to *P. alecto*, the region between *GABBR1* and *TCF19* of the *E. fuscus* MHC-I region was contracted (~1.1 Mb) compared to other eutherian mammals, including human (~1.7 Mb) and horse (~1.5 Mb).

In most mammals, MHC-I genes are located within three conserved duplication blocks, the α, κ and β blocks^26^,^27^, as highlighted in Fig. 2. The α block is located between *MOG* and *RNF39* and contains multiple MHC-I genes, including human leukocyte antigen A (*HLA-A*), in human, but is contracted in bats, horse and pig, and contains no MHC-I genes. The κ block, located between *TRIM26* and *ABCF1*, is also contracted in the bats but contains an expanded set of MHC-I genes in human, horse and pig. The β block is located in the region 3′ of *TCF19* and contains a set of duplicated MHC-I genes in human, horse and pig. In *P. alecto*, this region was not assembled but based on homologous gene architecture of the MHC-I region across eutherians, we propose that the MHC-I genes could be located in this region. As the bat genome was sequenced using short Illumina reads, assembly of highly complex and repetitive regions of the genome, such as those containing multiple MHC-I genes can be extremely difficult. Therefore, the presence of multiple class I genes within the region 3′ of *TCF19* may explain the failure of this region to assemble in the bat genome. The *E. fuscus* genome confirmed the absence of class I genes in the α and κ duplication blocks of bats and provided evidence for the presence of a β duplication block, with the identification of a single MHC-I gene located downstream from *TCF19* (Fig. 2). No class I genes were identified in the 179,283 bp of sequence available upstream of *GABBR1*. Eleven other class I genes were located on six separate scaffolds within the *E. fuscus* genome, indicating that it is possible that these genes are located outside the MHC-I region of this species (not shown).

### Identification, Sequencing, Assembly and Annotation of MHC-I Associated BAC Clones

As no complete MHC-I genes were identified in the assembled *P. alecto* genome and the MHC-I region could not be completely resolved using the genome sequence, a *P. alecto* BAC library was used to identify the remaining MHC-I region. Initial screening of the entire *P. alecto* BAC library with overgo probes corresponding to the conserved α3 domain of MHC-I genes and 13 MHC-I flanking framework genes, which span the MHC-I region of other mammals (Supplementary Table 1), yielded 92 BAC clones potentially corresponding to the MHC-I region. PCR was used to confirm the gene content of each of the clones, using primers specific for MHC-I and flanking framework genes used to screen the BAC library (listed in Supplementary Table 2), revealing that 49 out of the 92 BAC clones contained genes of interest. BAC end sequences were also determined for these 49 positive clones using Sanger sequencing to determine whether any of the BAC clones overlapped with scaffold555 from the genome or with one another. End sequences from two clones corresponded to scaffold555, with both mapping to the same region ~480 kb–~620 kb of scaffold555. BAC P56F16 was selected for next generation sequencing and confirmed the accurate assembly of the mapped region. Fingerprinting, using restriction enzyme digestion and pulsed-field gel electrophoresis, was employed to help identify BAC clones with unique banding patterns, thus eliminating the possibility of sequencing similar clones. A total of eight unique BAC clones, with different banding patterns, were finally selected for further sequencing and analysis.

**Figure 3:**
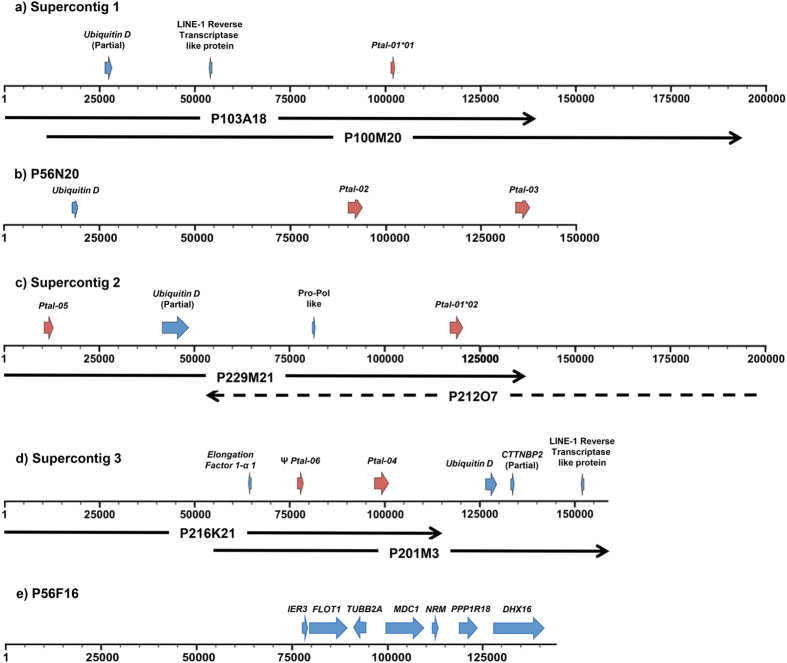
Organisation and gene content of MHC-I positive supercontigs and contigs: (**a**) Supercontig 1 (197,306 bp) comprised of BAC contigs P100M20 and P103A18, (**b**) BAC contig P56N20 (143,838 bp), (**c**) Supercontig 2 (154,408 bp) comprised of BAC contigs P212O7 and P229M21, (**d**) Supercontig 3 (192,498 bp) comprised of BAC contigs P201M3 and P216K21, (**e**) contig P56F16 (140,458 bp). Red arrows represent MHC-I genes and blue arrows represent other annotated genes. ^ψ^ represents putative pseudogenes. Transcriptional orientation of genes is indicated by the direction of the arrows.

The sequences obtained for the eight BAC clones assembled into individual contigs, six of which further assembled into three supercontigs. Genomic maps of the MHC-I gene containing contigs and supercontigs are illustrated in Fig. 3. Reference mapping of BAC clones back to the bat MHC-I genomic region confirmed that BAC clone P56F16 corresponded to a portion of scaffold555 (479,167 bp–619,625 bp; Fig. 1). Annotation of sequenced BAC clones was performed using GENSCAN^47^ to identify ORFs, followed by BLAST searches against the NCBI database. Coordinates of the predicted genes are shown in Table 2. Dotplot analyses were performed to compare each of the BAC supercontigs and contigs with one another to determine if any of the BACs represented haplotypes. These analyses revealed that supercontigs 1 and 2 were highly similar and likely represent haplotypes (Supplementary Fig. 1a). Supercontig 1 contains a single class I gene which is likely an allele of the class I gene at position 117,948bp–120666bp on supercontig 2. Supercontig 3 and scaffold P56N20 appeared to represent unique regions (Supplementary Fig. 1b–f). A total of six unique class I sequences were identified from the BAC contigs and were named *Ptal-01* through *Ptal-06*, with the two MHC-I alleles named Ptal-01*01 and *02. *Ptal* is an abbreviation of *P. alecto* with individual loci labelled with Arabic numerals (01 to 06) followed by an on-line asterisk to represent alleles (*01 and *02)^48^. For contigs with more than one MHC-I gene, the distance between the two genes ranged from 19 kb (between *Ptal-04* and *Ptal06*) – 104 kb (between Ptal-01*02 and *Ptal-05*), which is well within the range of 4.8 kb (between *HLA-K* and *HLA-U*) – 770 kb (between *HLA-E* and *HLA-C*) in the human MHC-I region^17^. Other non-MHC-I genes were also identified within the supercontigs and contigs including Uniquitin D (*UBD*), which was present on all three supercontigs and contig P56N20. All copies of *UBD* contained premature stop codons and are presumably pseudogenes. The locations of the seven MHC-I genes (including two alleles) identified on the BAC clones are shown in Fig. 3.

The BAC contigs and supercontigs were queried against the *P. alecto* whole genome sequence to confirm that no additional class I genes were present in the genome and to identify scaffolds that overlapped with the BAC contigs. The class I genes on the BAC contigs and supercontigs showed some similarity to the α3 domains of partial class I genes identified in contig C18352586 and scaffold7320 but did not otherwise overlap. The BAC contigs and supercontigs also showed similarity to scaffolds in the genome that contained non MHC-I genes including *UBD* and elongation factor 1α. However, these scaffolds did not contain class I genes and did not overlap with the BAC contigs or supercontigs.

### Chromosome Location

**Figure 4:**
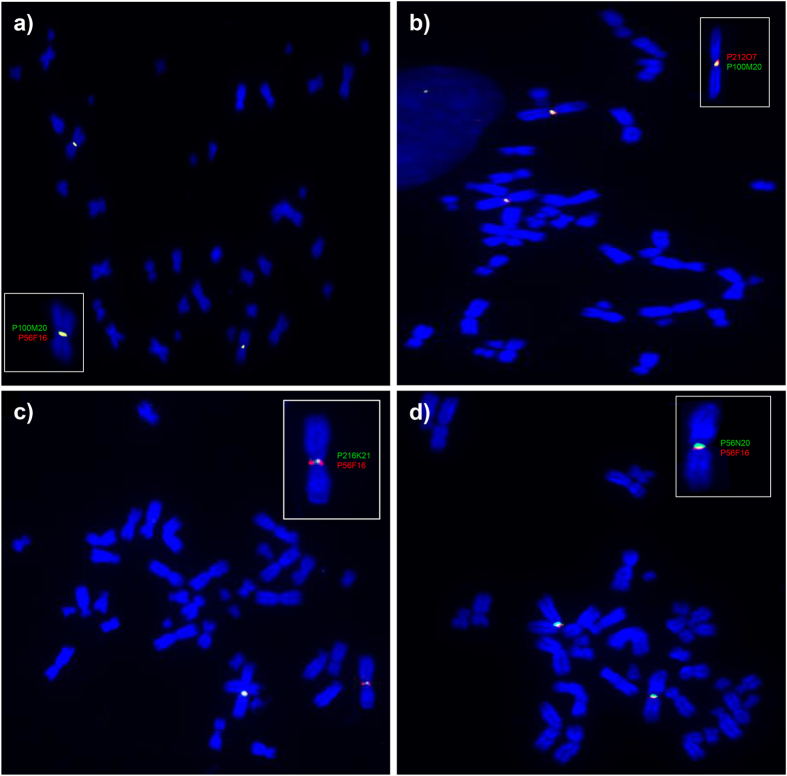
Fluorescence *in situ* hybridization (FISH) of MHC-I containing bat BAC clones on bat metaphase chromosomes. (**a**) Co-localization of BACs containing Ptal-01*01 (P100M20) (green), to the MHC-I region on scaffold555 (P56F16) (red). (**b**) Co-localization of BACs containing Ptal-01*01 (P100M20) (green), with Ptal-01*02 and *−05* (P212O7) (red). (**c**) Co-localization of BACs containing *Ptal-06* and *−04* (P216K21) (green) with the MHC-I region on scaffold555 (P56F16) (red). (**d**) Co-localization BACs containing *Ptal-02* and *-03* (P56N20) (green) with the MHC-I region on scaffold555 (P56F16) (red).

For chromosome co-localisation, BAC P56F16 was used as a reference for the bat MHC-I region identified in the bat genome with other MHC-I containing BAC clones. All eight BAC clones positive for MHC-I and related genes localised on the same male bat chromosomes using fluorescence *in situ* hybridisation (FISH) with reference to BAC clone P56F16 (Fig. 4). Our results support the co-localisation of the MHC-I positive BAC clones with the genomic region identified in the *P. alecto* genome. BAC clones representing supercontigs 1 and 2 (corresponding to Ptal-01*01, −01*02 and *−05*) clearly overlapped with the genomic reference clone (Fig. 4a,b). Although the locations of supercontig 3 (*Ptal-04* and *−06*) and clone P56N20 (*Ptal-02* and *−03*) relative to the genomic scaffold are more difficult to determine due to the intensity of the fluorescent signal, both appear to be on the same side of the centromere as the reference clone (Fig. 4c,d). The overlap of supercontig 3 and the genomic reference scaffold is most visible on the chromosome outside the boxed area (Fig. 4c). Similarly, overlap in the signals from BAC P56N20 and the genomic reference clone is evident on the two chromosomes with a fluorescent signal. However, the chromosome that is bent at the centromere illustrates most clearly that both clones are on the same side of the centromere (Fig. 4d). By comparing chromosome size and morphology against previously published karyotyping data for *P. alecto*^49^, the bat MHC-I region is likely to be located on chromosome 1.

### Promoter Analysis of the Bat MHC-I Genes

**Figure 5:**
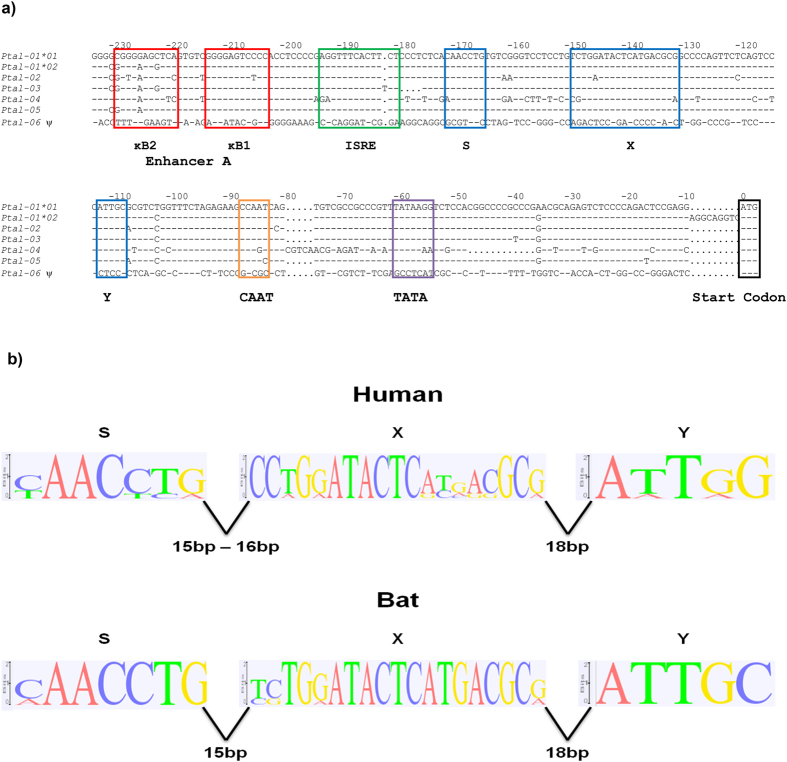
Promoter element analysis of bat MHC-I genes. (**a**) Putative promoter elements of bat MHC-I genes. Boxed sequences indicate putative sites of Enhancer A (red), interferon stimulated response element (ISRE; green), S-X-Y motifs (blue), CAAT (orange) and TATA (purple) binding boxes and start codon (black). ^ψ^represents putative pseudogene. (**b**) Comparison of the S-X-Y motifs in MHC-I genes of bat and human. Logos of corresponding position-specific scoring matrix models are presented. The height, of each stack of symbols (y-axis) in log_2_ scale represents information content in each position of the DNA sequence (x-axis), with a maximum value of 2.

Transcription of class I genes is tightly regulated by promoter elements upstream of the transcription start site, including enhancers, response elements and various binding boxes. The region 235 bp upstream of the translation start site of the seven bat MHC class I genes (Ptal-01*01, −01*02, *−02*, *−03*, *−04*, *−05*, and *−06*) were analysed to identify putative promoter elements. Using manual examination and annotation, with reference to other mammalian promoter elements, Enhancer A (both κB1 and κB2 binding sites), interferon stimulated response element (ISRE), S-X-Y motif, CAAT and TATA binding boxes, were identified with minimal variation in all loci with the exception of *Ptal-06* (Fig. 5a). The Y motif was the most conserved amongst the promoters, with no nucleotide variation across the set of genes analysed. Promoter elements were not found in the *Ptal-06* locus, consistent with it being a pseudogene. Table 3 summarises the coordinates of bat MHC-I S-X-Y motifs within the BAC contig and supercontigs. The bat S-X-Y motif was further analysed in the six bat MHC-I genes with comparison to S-X-Y motifs of six HLA genes (*HLA-A*, *-B*, *-C*, *-E*, *-F* and *-G*), producing a sequence logo diagram (Fig. 5b). The distance between each motif (S and X, X and Y) was highly conserved between bat and human. The bat S-X-Y motif also appears to be more conserved between the bat MHC-I genes compared to those of the human HLA genes.

### Sequence and Phylogenetic Analysis of the Bat MHC-I Genes

To date, a total of seven MHC-I genes have been identified in *P. alecto*: Ptal-01*01, −01*02, −02, *−03*, *−04*, −*05* and *−06*. An alignment of the deduced protein sequences of the seven bat MHC-I genes with three classical (*HLA-A*, *-B* and *-C*) and one non-classical (*HLA-G*) human MHC-I gene is shown in Supplementary Fig. 2. Of the seven predicted bat class I coding sequences, *Ptal-06* appeared to be a processed pseudogene due to the presence of a fused leader peptide and α1 domain and the absence of a complete α3 domain. Processed pseudogenes are generated through retrotransposition of partial or complete cDNA copies of corresponding mRNA transcripts, or even the mRNA itself, back into the genome. Once integrated, the mRNA sequence is replaced by its DNA equivalent during the next replication cycle, followed by repair and ligation^50^,^51^,^52^. The bat MHC-I sequences contain many of the features conserved in class I sequences from other mammals. These include cysteine residues in the α1 and α2 domains, which are likely to form intra-chain disulphide bonds, the β_2_M interaction sites, CD8 co-receptor interaction sites and glycosylation sites. The putative NK receptor-binding region was also identified and was highly variable among the bat class I genes, similar to other species. All putative interaction sites were predicted based on human *HLA* and are conserved across mammals and non-mammals^53^,^54^,^55^. A number of unpaired cysteine residues were present in the *P. alecto* MHC-I molecules. *Ptal-02* contained unpaired cysteine residues in the α1 domain at position 97 and in the α3 domain at position 275. *Ptal-05* contained an unpaired cysteine in its α1 domain at position 97 and the cytoplasmic domain at position 393. *Ptal-06* and –01*02 each contained an unpaired cysteine in their α2 domain at position 151 and 203 respectively.

**Figure 6:**
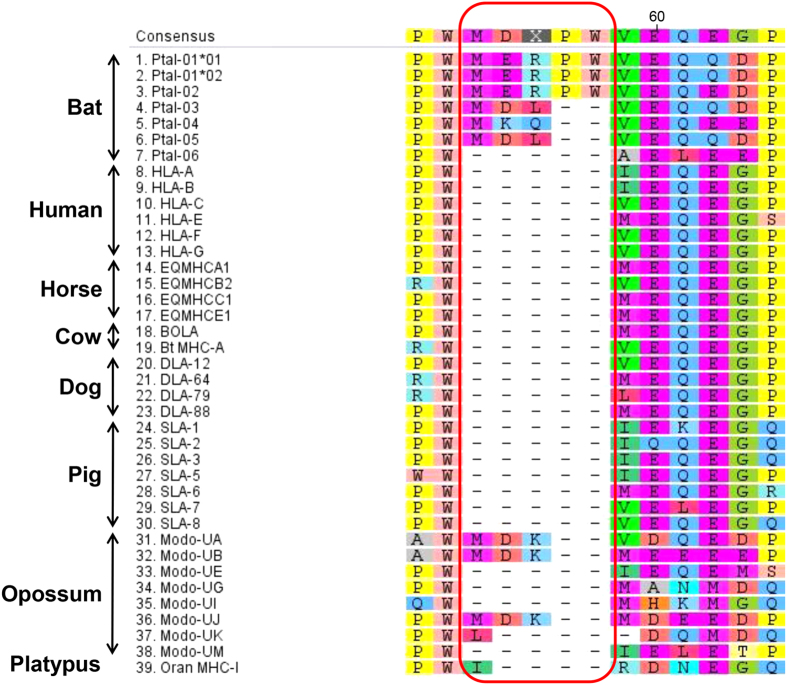
Unique amino acid residue insertions in the α1 domain of bat MHC-I sequences (circled in red).

Considerable length variation was also observed among the bat class I genes and three MHC-I variants were identified based on the presence of unique insertions within the PBG compared to human MHC-I genes (Supplementary Fig. 2). Three of the bat MHC-I loci (Ptal-01*01, −01*02 and *−02*) contained a 5-aa insertion between residues 78 to 82 of the α1 domain and three (*Ptal-03*, *−04* and *−05)* contained a 3-aa insertion in the same region. Only the putative pseudogene *Ptal-06* contained no amino acid insertions within this region. Comparison of the bat class I genes against those from a variety of other mammals demonstrated that the 5-aa insertion is unique to the bat sequences while the 3-aa insertion is present only in bat and opossum MHC-I genes (Fig. 6). Furthermore, the α1 domain of *Ptal-04* was 26-aa longer than all other mammalian MHC-I genes and the cytoplasmic domain of the *Ptal-05* locus contains a 17-aa insertion (Supplementary Fig. 2). Previously described MHC-I transcriptome sequences from *P. alecto* were also compared with our genomic loci. As the transcriptome sequences were from pooled tissues from multiple individuals, it is impossible to distinguish loci from alleles at this stage. All seven partial MHC-I transcripts contained the unique three amino acid insertion within their PBG (Supplementary Fig. 3b). Based on phylogenetic analysis, four transcripts (Locus23_971_Transcript_3/5, Locus25_954_Transcript_1/8, Locus27_2413_Transcript_4/9 and Locus31mer_4890_Transcript_1/2) clustered closely to *Ptal-04*, confirming the transcription of the 3-aa variants^42^ (Supplementary Fig. 3a). The remaining three transcripts (Locus23_2912_Transcript_1/1, Locus25_954_Transcript_6/8 and Locus25_954_Transcript_8/8) do not correspond with any of the loci identified in the genome (Supplementary Fig. 3), indicating that additional class I genes not identified in the BAC clones likely exist within the *P. alecto* genome.

Sequence similarity at both the nucleotide and deduced amino acid level was compared between the six bat MHC-I loci (excluding the *Ptal-06* locus) across the α1–α3 domains and is shown in Supplementary Table 4. Overall, the bat MHC-I genes have nucleotide and amino acid sequence similarity of 86–95% and 78–90% respectively. Bat MHC-I genes have higher conservation compared to those from human, horse, pig and dog, with nucleotide and amino acid sequence similarity within each species, ranging between 75–92% and 64–87% respectively (data not shown). In order to include the putative pseudogene *Ptal-06* in the analysis, only the α1–α2 domains were analysed across all seven bat MHC-I loci (Supplementary Table 5). A wider range of nucleotide and amino acid sequence similarity of approximately 70–93% and 54–87% respectively was observed across the α1–α2 domains. The α1–α2 domains of *Ptal-06* is also highly divergent from other mammals, sharing only 52–75% and 33–61% nucleotide and amino acid sequence similarity respectively (data not shown).

**Figure 7:**
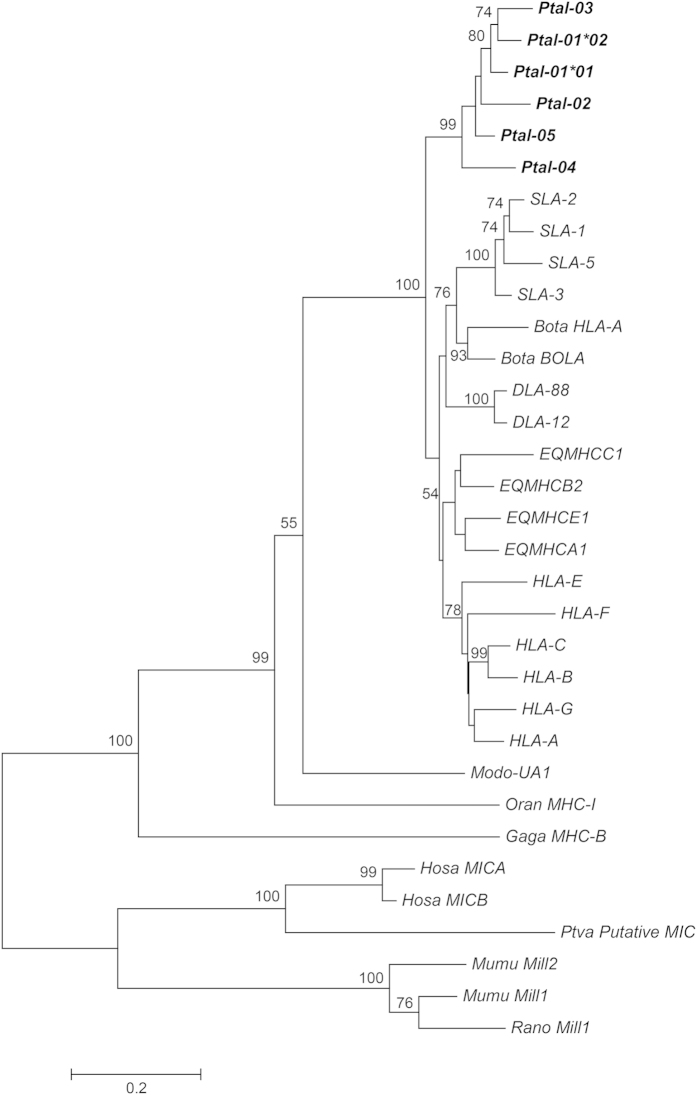
Phylogenetic tree of MHC-I genes. Maximum likelihood phylogeny was used based on alignment of nucleotide sequences to exons 2, 3 and 4, corresponding to α1, α2 and α3 domains respectively. GTR with discrete Gamma distribution was used to model evolutionary rate differences among sites (5 categories ( + *G*, parameter = 1.5470)). The tree is drawn to scale, with branch lengths representing the number of substitutions per site. Branch support is indicated as percentage of trees out of 1000 bootstrap replicates that produce the same branching order. *SLA* – swine leucocyte antigen; *EQMHC* – equine MHC; *DLA* – dog leucocyte antigen; *HLA* – human leucocyte antigen; *Bota* – *Bos taurus*; *Modo* – *Monodelphis domestica*; *Oran* – *Ornithorhynchus anatinus*; *Gaga* – *Gallus gallus*; *Hosa* – *Homo sapiens*; *Ptal* – *Pteropus alecto*; *Ptva* – *Pteropus vampyrus*; *Mumu* – *Mus musculus*; *Rano – Rattus norvegicus*.The non-classical MHC-like, human *MIC* and rodent *Mill*, genes were used as outgroups. A putative *MIC* ortholog from the *P. vampyrus* genome was also included in the analysis.

Phylogenetic analysis was performed using nucleotide sequences from exons 2, 3 and 4, corresponding to α1, α2 and α3 domains, of the six bat MHC-I genes with the corresponding region of sequences from other vertebrates. The putative pseudogene, *Ptal-06*, was excluded from the analysis as it lacks the α3 domain. As shown in the Maximum Likelihood (ML) tree in Fig. 7, the bat and non-bat MHC-I genes cluster in a species-specific manner consistent with the orthologous origin of MHC-I genes. Similar results were obtained when Neighbour Joining (NJ)^56^ and Minimum Evolution (ME)^57^ methods were employed (Supplementary Fig. 4a,b respectively). Separate phylogenetic analysis of the MHC-I hyper-variable regions, α1 and α2, and the highly conserved α3 domain produced similar results (Supplementary Fig. 4c,d respectively).

### 3D Protein Modelling of the Bat MHC-I Genes

**Figure 8:**
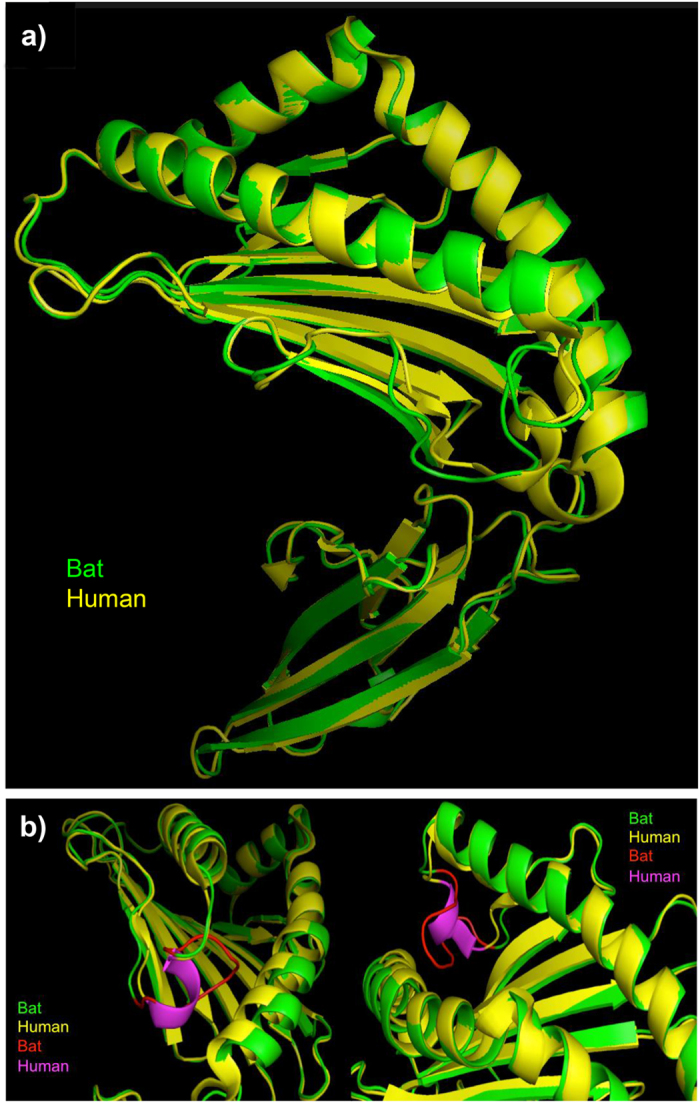
Predicted protein models of the α1 and α2 domains of bat MHC-I genes. (**a**) Overlay of the predicted *Ptal-01* protein model (green) with crystallographic *HLA-B* protein model 3LN4 (yellow), downloaded from the Protein Data Bank, PDB. (**b**) A zoomed-in view of the area with structural difference highlighted in red (bat) and magenta (human), due to the 5 amino acid residue insertion in the bat α1 domain, demonstrating the rigid α-helix structure of the human MHC-I molecule and the flexible coils and turns produced by the bat MHC-I.

To determine whether the unique 3- and 5-aa insertions in the α1 domain of the bat class I genes affects their 3D conformation, structural analysis was performed using *Ptal-01* and *-03* as representative sequences for the variants containing the 5- and 3-aa insertions respectively. The predicted crystal structures were determined based on the human class I gene *HLA-B* (3LN4) for *Ptal-01* and macaque class I gene (3JTS) for *Ptal-03* using CPHmodels. Similar 3D models were obtained for the predicted structures of for *Ptal-01* and *-03* (see Supplementary Fig. 5). The predicted *Ptal-01* model was further compared with human *HLA-B* (3LN4) model by superimposing the predicted bat protein onto the human *HLA-B* crystal structure (Fig. 8). A similar analysis was performed using *Ptal-03* (data not shown). The additional five amino acid residues present in the PBG of the bat class I gene resulted in a structural change from a rigid α-helix structure of conventional MHC-I molecules to flexible coils and turns. As shown in Fig. 8, the human MHC-I molecule has the α-helical structure (magenta) while the bat MHC-I molecule consists of relaxed coils and turns (red). Although the presence of rigid proline residues flanking the two ends of this region (residues 52 and 64 in Fig. 6) could hamper its flexibility, a longer, relaxed peptide structure could potentially circumvent this issue. If the bat class I molecules are classical in nature, the predicted change in structure based on modelling with *HLA-B* could potentially bestow more flexibility to the end of the PBG and allow it to accommodate a larger and/or more diverse repertoire of antigens. Homology modelling of *Ptal-01* was also performed using the I-TASSER method^58^. Protein structures from the PDB that were closest to the predicted *Ptal-01* models were the human class Ia and Ib molecules, *HLA-B*, *HLA-C* and *HLA-E,* and the mouse class Ia molecule, *H-2K*, consistent with *Ptal-01* being closely related to class Ia molecules.

Root mean square deviations (RMSD) of all predicted bat MHC-I molecules were determined against a reference model (3LN4) and their individual query models (3JTS for *-03*). Other resolved and predicted models from various vertebrates were included in Supplementary Table 6 for comparative purposes. The RMSD is a measure of the average distance between atoms of superimposed proteins. The low RMSD values of the predicted bat MHC-I molecules indicate high confidence for the overall predicted structure^59^. However, crystallography of the actual bat MHC-I molecules will be required to confirm our predictions.

## Discussion

This study represents the first analysis of the MHC-I region of any Chiropteran species, filling an important phylogenetic gap in understanding the evolution of the mammalian MHC and is the first step in determining the role of MHC-I molecules in viral infection in bats. Our partial map of the *P. alecto* MHC-I region is highly conserved in gene architecture but MHC-I genes are absent from at least two of the three duplication blocks within the MHC-I region. This architecture was confirmed in a second bat species, *E. fuscus,* which contained only a single class I gene within its β duplication block. Additionally, several *P. alecto* MHC-I genes contain unique insertions within the PBG, potentially reflecting non-classical roles or affecting antigen binding which in turn may contribute to the ability of bats to control viral infections.

To our knowledge, this is the first attempt at resolving the highly repetitive MHC-I region of any species solely employing next generation sequencing (NGS) technology using a combination of the recently completed bat genome and BAC sequencing. A single scaffold of 927,264 bp, containing a partial MHC-I region corresponding to the extended and classical class I subregions, was identified in the *P. alecto* genome^12^. This region contained a conserved framework structure flanked by *OR2H3* and *TCF19* but did not include any MHC-I genes. The corresponding region of a second bat species, *E. fuscus*, contained a similar architecture and was ~1.1Mb in size. The MHC-I region identified in the bat genome is highly contracted compared to the same region in other mammals including human and horse, which span ~1.7 Mb and ~1.5 Mb respectively^17^,^21^. The pig (*S. scrofa*) is the only other mammal with a contracted MHC-I region, with the corresponding region spanning just over 1 Mb in length^25^,^60^. Although the size of the MHC-I region in the genome of *P. alecto* remains to be determined, the entire MHC-I region of *E. fuscus* from *GABBR1* to the MHC-I gene is only ~1.2Mb. The smaller size of the bat MHC-I region is consistent with the smaller genome size of bats, estimated to be ~2.0 gigabases (Gb) compared to humans and other mammals, which have an average genome size of ~3.5 Gb^12^,^61^.

In terms of genetic content and gene organisation, the partial *P. alecto* MHC-I region and the corresponding region of *E. fuscus* remain highly conserved with that of other mammals. Important framework genes including *MOG*, protein phosphatase 1 regulatory subunit 11 (*PPP1R11*), *TRIM26*, *TRIM39*, guanine nucleotide-binding protein-like 1 (*GNL1*) and *TCF19*, are present in the bat MHC-I region. These highly conserved MHC-I framework genes and their ordered organisation represent the ancestral mammalian structure^26^. In other eutherian species, the region between *MOG* and *TCF19* contains two duplication blocks, α and κ, which each contain class I genes. Unlike all other eutherian mammals sequenced to date, no MHC-I genes were found within the α or κ duplication blocks in the genomes of either *P. alecto* or *E. fuscus*^15^,^19^,^23^,^27^.

BAC clones containing MHC-I and framework genes were sequenced in an attempt to identify the remaining *P. alecto* class I region, and determine the number and organisation of MHC-I loci in this species of bat. Although we were unable to obtain a complete, contiguous map of the MHC-I region, analysis of the chromosomal location of the BAC clones using FISH confirmed that they co-localised with the MHC-I region identified in the *P. alecto* genome. The most likely location for the MHC-I genes identified to date is downstream of *TCF19*, corresponding to the β duplication block. This is in agreement with the MHC-I region of *E. fuscus* which contains a single MHC-I gene downstream of *TCF19* within the β duplication block.

Flanking genes identified in the *P. alecto* BAC contig and supercontigs were also atypical compared to those present in the MHC-I region of humans and other species. In humans, the β duplication block is demarcated by two highly conserved flanking genes *MICB* and *PSORS1C3*, and contains two classical class I genes (*HLA-B* and *-C*), various HLA complex non-protein coding RNA genes and multiple pseudogenes, including ubiquitin specific peptidase 8 pseudogene 1 (*USP8P1*), ribosomal protein L3 pseudogene 2 (*RPL3P2*), WAS protein family member 5 pseudogene (*WASF5P*) and fibroblast growth factor receptor 3 pseudogene (*FGFR3P*)^17^,^27^. In the *P. alecto* MHC-I region, a partial *UBD* (possibly an ubiquitin pseudogene) is present between Ptal-01*02 and *−05* in supercontig 2, but no *MIC* or *PSORS* genes were identified in any of the contigs or supercontigs. Furthermore, only a putative pseudogene of *MICB* was identified on a small scaffold in the *P. alecto* genome. Orthologues of *MIC* or *Mill* were also absent from transcriptome data obtained from *P. alecto* immune tissues and stimulated cells^42^. These genes were also absent from the MHC-I region of *E. fuscus*. In supercontig 3, elongation factor 1-α 1 and *UBD* flanked the two class I genes, *Ptal-06* and *−04*. Elongation factor 1-α 1 is not present within the MHC of other species but *UBD* is usually located at the 5′ end of the α duplication block, adjacent to *GABBR1* in the MHC-I region in all species examined, including *E. fuscus*. In the *P. alecto* genomic scaffold, there was insufficient sequence upstream of *GABBR1* to detect evidence of *UBD*. Furthermore, there was no evidence of *GABBR1* in the *P. alecto* supercontigs, indicating that it is unlikely that the *P. alecto* MHC-I genes are located at this 3′ end of the MHC-I region. Information from the *E. fuscus* MHC-I region also supports our hypothesis that at least some *P. alecto* MHC-I genes are located in the β duplication block. However, as the two suborders of bats have evolved independently since their divergence approximately 68 mya, final confirmation of the nature of the *P. alecto* MHC-I region may await additional sequence information, for example from PacBio sequencing which is likely to provide higher resolution of complex regions such as the MHC-I region.

### Duplication of MHC-I Genes within the MHC Region may have occurred in a Step-wise Manner in Eutherian Mammals

**Figure 9:**
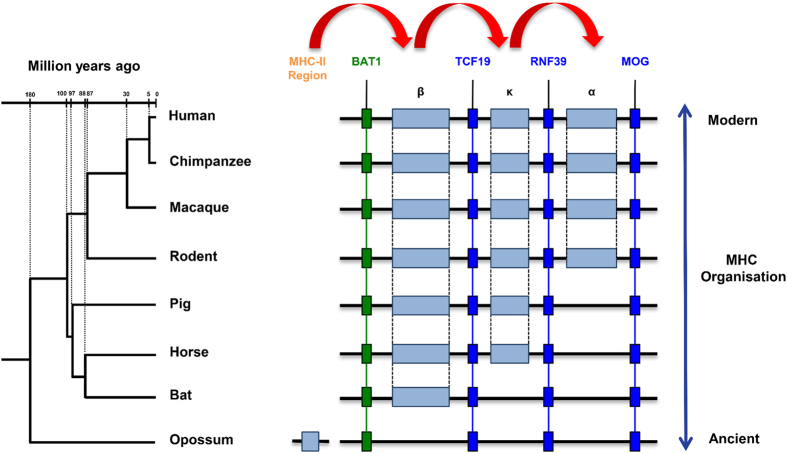
Proposed step-wise duplication of the MHC-I genes. MHC-I genes first migrated into the β duplication block of the MHC-I region, followed by subsequent step-wise duplication into the κ duplication block, and finally into the α duplication block. Individual species-specific expansion and contraction of MHC-I genes occur within the duplication blocks.

Kumanovics *et al.*^19^ offered two alternative explanations for the evolution of the mammalian MHC-I region within the highly conserved framework structure. Class I genes may have been present in all three class I duplication blocks in the mammalian ancestor and class I genes were lost in a species specific manner. Alternatively, class I gene expansion may not have occurred in all of the permissive sites in some species such as pigs and other Laurasiatherian mammals. The bat MHC-I region provides a link between the ancestral genome of marsupials with that of eutherian mammals. Based on comparative analysis of bats with other mammals, we present a model for the evolution of the MHC-I region in eutherian mammals in which MHC-I genes duplicated in a stepwise manner across the MHC-I region. We propose that MHC-I genes first originated in the hybrid class I/II region as previously observed in the MHC regions of marsupials, monotremes and other lower vertebrates^18^,^20^,^29^,^62^, followed by subsequent translocation into the MHC-I region in eutherian mammals after the divergence of marsupials and eutherians. Within the eutherian lineage, we propose that MHC-I genes duplicated in the β, κ and α blocks in a stepwise manner. Class I genes first translocated into the β duplication block in the bat and other mammals, followed by subsequent translocation and duplication into the κ block as demonstrated in horse and pig, and into the α duplication block for some other eutherian lineages including primates and rodents (Fig. 9). The absence of partial MHC-I genes or pseudogenes in the α or κ duplication blocks in two bat MHC-I regions further supports our hypothesis for a single-block origin for MHC-I genes within the MHC-I region of eutherian mammals. However, further examination of the MHC-I region from additional bat species and from other eutherian mammals, such as elephants (Afrotheria) and armadillos/sloths (Xenarthra) will be important in confirming our hypothesis and for determining the nature of translocation of class I genes across the framework structure of the MHC-I region.

### Bats have a closely related repertoire of MHC-I Genes

Papenfuss *et al.*^42^ previously described seven MHC-I transcripts from tissues and cells pooled from multiple individuals. Sequence and phylogenetic analysis of genomic and transcriptome sequences provide further evidence that *P. alecto* has a more closely-related MHC-I repertoire compared to other eutherians, an observation which is striking given the observed heterozygosity of the bat genome^12^. Conservation of the promoter regions of the *P. alecto* genomic loci also revealed high conservation of S-X-Y motifs between loci consistent with the possibility that all of the genomic class I loci identified to date are either classical or non-classical in nature. Further investigation will be required to determine the nature of the bat class I genes to determine the number of classical and non-classical class I genes in bats.

### Significance of 5-aa Insertion in the Bat MHC-I PBG

Unique insertions within the PBG of the bat MHC-I genes hint at differences in the peptide binding capability of bat MHC-I molecules or differences in function associated with non-classical roles. Classical MHC-I molecules are generally capable of presenting processed peptide antigens of 8–11 aa in length^36^,^37^,^38^,^39^. Only in rare occurrences are longer antigens up to 25-aa in length presented^63^,^64^. These large antigens bulge out of the PBG, affecting the 3D topography of the antigen interaction site with the T cell receptors (TCR). Varying antigen lengths not only affect the outcome of TCR/peptide-MHC-I engagement^65^, but also the control of CD8^+^T cell responses^66^. With the discovery of unique 5-aa insertions within the MHC-I PBG, bats could potentially present antigens longer than the “prescribed optimal” length due to the presence of a more flexible PBG end. If bat MHC-I molecules preferably present larger antigens, there could be profound implications for the diversity of the antigen repertoire presented, the efficiency of peptide loading/ antigen presentation and the nature of the TCR-peptide-MHC complex. Elucidating the diversity of peptides presented by bat MHC-I molecules and the crystal structure of the PBG and TCR-peptide-MHC complex will be required to determine the nature of antigen presentation by bat MHC-I molecules. Although modelling predictions suggest that *Ptal-01* may be functionally similar to human and mouse class Ia molecules, it is also possible that some or all of the identified bat MHC-I molecules are non-classical in nature and play roles other than antigen presentation. Many of the well-studied human and mouse class Ib genes have adaptations to their PBG to accommodate different functions. For example, *CD1* molecules have narrow but deeper peptide binding grooves and present lipid antigens to T cells while *HFE* has a closed PBG and interacts with transferrin to regulate iron uptake^41^.

## Conclusion

The bat MHC-I region fills an important phylogenetic gap in the evolution of the mammalian MHC-I region. Comparative analysis of the *P. alecto* MHC-I region with other mammals led us to hypothesise a step-wise duplication process of MHC-I genes within the eutherian class I region. The identification of *P. alecto* class I molecules containing unique PBGs could potentially increase the efficiency and diversity of viral antigen presentation by the bat’s immune system. Further studies linking the uniqueness of bat MHC-I molecules and the ability of bats to control viral replication and coexist with viruses is highly anticipated.

## Materials and Methods

### *P. alecto* Genome Data and Annotation

The recently completed *P. alecto* genome was interrogated for MHC-I genes and conserved class I flanking genes using BLAST searches^67^. Scaffolds containing MHC-I flanking genes were re-annotated manually using GENSCAN^47^ for gene prediction and their identity confirmed using BLAST^67^ against the NCBI database.

### BAC Screening, Sequencing and Analysis

A *P. alecto* BAC library was commercially constructed by Amplicon Express (Washington, USA) using genomic DNA extracted from the liver of a wild caught adult male bat. BAC clones contained inserts with an average size of ~130 kb cloned into the CopyControl™ pCC1BAC™ vector^68^. The BAC library consisted of 92,160 clones, representing approximately 5 fold coverage of the *P. alecto* genome. All animal experiments were approved and carried out in accordance with the guidelines by the Australian Animal Health Laboratory (AAHL) animal ethics committee (protocol 1389). The BAC library was screened with overgoes specific for MHC-I and MHC-I flanking framework genes (Supplementary Table 1). Overgoes were designed using overgo maker (http://bioinf.wehi.edu.au/cgi-bin/overgomaker) using sequences identified in the *P. alecto* whole genome.

Overgo probes were labelled with ^32^P dCTP using the Prime It II labelling kit (Stratagene) following the manufacturer’s instructions. High density BAC library filters were hybridised overnight with pools of radioactively labelled overgoes in Church buffer (7% SDS, 1% bovine serum albumin, 1mM EDTA, Na_2_HPO_4_ 0.25 M, pH 7.2) at 65 °C. Positive clones were further screened by PCR using gene-specific primers (Supplementary Table 2). Positive clones were then restriction digested using 10U of *Hind*III incubated at 37 °C for 4 h to determine their fingerprinting patterns. Pulsed field gel electrophoresis was then employed to visually resolve the restriction digested BAC clones, using 1% Pulsed Field Certified Agarose in 0.5x TBE, on the CHEF-DR^®^ III System (Bio Rad), together with a Cooling Module, Variable Speed Pump and Electrophoresis Cell. Samples were then run for 13h at 14 °C at a voltage of 6 V/s, 120° field angle, an initial time of 1 s and final time of 20 s. Unique banding patterns of individual clones were used to select the candidate for NGS.

Single end sequencing libraries were constructed using the GS FLX Titanium Rapid Library Preparation Kit (GS FLX + Series – XL + ; Roche) on selected clones, which were subsequently sequenced using the Roche 454 platform with the FLX + long read chemistry (Roche). BAC end sequencing was also performed on all clones using Sanger sequencing with CopyControl™ pCC1BAC™ vector sequencing primers pCC1™-F (5′-GGATGTGCTGCAAGGCGATTAAGT TGG-3′) and pCC1™-R (5′-CTCGTATGTTGTGTGGAATTGTGAGC-3′).

Raw reads were filtered, trimmed and assembled using a combination of CLC Genomics 6.5.2 (CLC bio, Aarhus, Denmark), Clone Manager 9.0 (Sci-Ed Software, Morrisville, USA) and SeqMan Pro 11.2.1 (DNASTAR^®^, Madison, USA) software. ORFs in contigs and supercontigs were predicted using GENSCAN^47^ and their identity confirmed using BLAST^67^ against the NCBI database. Further manual annotation was performed to confirm and obtain the full-length MHC-I genomic sequences.

### Fluorescence *In Situ* Hybridisation (FISH)

FISH was employed following the protocol described previously^69^, with some modifications. Briefly, metaphase chromosome spreads were prepared from male *P. alecto* primary kidney cells^70^. DNA (1 μg) from each BAC clone isolated (Supplementary Table 3) was labelled by nick translation with Green-dUTP or Orange-dUTP (Abbott Molecular, U.S.A). 0.5–1.0 μg labelled BAC DNA, co-precipitated with 1 μg of *P. alecto* sheared genomic DNA, was hybridised to metaphase chromosomes and fluorescent signals were detected following the protocol described previously^69^. A Zeiss Axio ScopeA1 epifluorescence microscope was used to visualise fluorescent signals. Images of fluorescent signals and DAPI-stained metaphase chromosomes were captured on an AxioCam MRm Rev.3 CCD (charge-coupled device) camera (Carl Zeiss Ltd, Germany) and merged using Isis FISH Imaging System version 5.4.11 (MetaSystems, Germany).

### Comparative Analysis of Bat MHC-I Region and Genes

The human (*Homo sapiens*), horse (*Equus caballus*) and pig (*Sus scrofa*) MHC-I regions from the Ensembl annotation (versions GRCh37.p11 for human, EquCab2 for horse and Sscrofa10.2 for pig) were used for comparative analysis with the bat (*P. alecto*) MHC-I region using EasyFig software^71^. Bat genomes also used for comparative analysis include the *P. vampyrus* genome (Ensemble, pteVam1) and the big brown bat, *E. fuscus* genome (GCA_000308155.1).

### Promoter Analysis

The region 600 bp upstream of human MHC-I genes (*HLA-A*, *-B*, *-C*, *-E*, *-F* and *-G*) was retrieved from Ensembl (version GRCh37.p11). The corresponding region of the bat MHC-I genes was retrieved from the bat BAC clone sequences. The promoter regions of the bat class I genes were analysed by comparison to the human genes. All sequences upstream from the start codon were manually analysed using Clone Manager to identify putative promoter elements: Enhancer A, Interferon Stimulated Response Element (ISRE), S-X-Y motifs, CAAT box and TATA box. Sequences were then collated and aligned, with sequence logos^72^ of the S-X-Y motifs illustrated using the Geneious version R7 software package created by Biomatters (Available from http://www.geneious.com/).

### Gene and Phylogenetic Analysis

MEGA software version 5.2.1^73^ was used for all gene and phylogenetic analysis. Bat MHC-I sequences were first aligned with human HLA sequences as reference using MUSCLE. Corresponding aligned nucleotide sequences were then subsequently used for phylogenetic analysis using the Maximum likelihood (ML) General Time Reversible (GTR) or ML Hasegawa-Kishina-Yano (HKY) model with discrete Gamma distribution and 1000 bootstrap replications^74^,^75^,^76^. The “Find Best Model (ML)” function was used to determine the appropriate substitution models for each dataset. The model with the lowest Bayesian Information Criterion (BIC) score is considered to best describe the substitution pattern for that dataset and was subsequently chosen for phylogenetic analysis. Neighbour Joining (NJ)^56^ and Minimum Evolution (ME)^57^ trees, with 1000 bootstrap replications, were also constructed to corroborate with the ML trees. Tree Explorer was used for tree visualisation and illustration. Base-By-Base^77^ was used to determine nucleotide and amino acid sequence identity between the different bat MHC genes identified to date.

### Structural Prediction and Protein Modelling

Selected bat MHC-I sequence structures were submitted to CPHmodels 3.2 Server for protein model prediction and reference templates were selected based on profile-profile alignment guided by secondary structure and exposure predictions^78^. The PDB ID of protein structures used as reference for model prediction of MHC-I molecules are listed in Supplementary Table 7. Predicted models were then analysed using the PyMOL Molecular Graphics System Version 1.5.0.4 by Schrödinger, LLC (Available from http://www.pymol.org/) with known protein models as reference. Structure prediction were also performed using the I-TASSER method^52^. Root mean square deviation (RMSD)^59^ was calculated by aligning and overlaying bat models against known reference models in PyMOL. All known reference protein models were downloaded from the Protein Data Bank.

### Data access

The *P. alecto* BAC contigs and supercontigs have been submitted to the GenBank database under their respective accession numbers: P56F16 (KP862824); P56N20 (KP862825); Supercontig 1 (KP862826); Supercontig 2 (KP862827); Supercontig 3 (KP862828).

The GenBank (http://www.ncbi.nlm.nih.gov/Genbank) accession numbers and Ensembl (http://asia.ensembl.org/index.html) transcript ID for the genes and gene products discussed in this paper are scaffold555 (KB030712.1); *Bos taurus* BOLA (BC109586); *B. taurus* HLA-A (BT020991); *Sus scrofa* SLA-1 (DQ992492); *S. scrofa* SLA-2 (AB231907); *S. scrofa* SLA-3 (AF464010); *S. scrofa* SLA-5 (NM_001114056); *S. scrofa* SLA-6 (AF464007); *S. scrofa* SLA-7 (AY463541); *S. scrofa* SLA-8 (AY463542); *Canis lupus familiaris* DLA-12 (CFU55026); *C. l. familiaris* DLA-64 (CFU55027); *C. l. familiaris* DLA-79 (Z25418); *C. l. familiaris* DLA-88 (CFU55028); *Equus caballus* EQMHCA1 (X71809); *E. caballus* EQMHCB2 (X79891); *E. caballus* EQMHCC1 (X79893); *E. caballus* EQMHCE1 (X79894); *Homo sapiens* HLA-A (NM_002116); *H. sapiens* HLA-B (NM_005514); *H. sapiens* HLA-C (NM_002117); *H. sapiens* HLA-E (NM_005516); *H. sapiens* HLA-F (NM_018950); *H. sapiens* HLA-G (NM_002127); *Monodelphis domestica* Modo-UA1 (NM_001044223); *M. domestica* Modo-UB (NM_001079820);*M. domestica* Modo-UE (NM_001171835); *M. domestica* Modo-UG (NM_001079813); *M. domestica* Modo-UI (NM_001171837); *M. domestica* Modo-UJ (NM_001171836); *M. domestica* Modo-UK (EU886706); *M. domestica* Modo-UM (EU886712) *Ornithorhynchus anatinus* MHC-I (AY112715);*Gallus gallus* MHC-B (ENSGALT00000000081); *H. sapiens* MICA-001 (ENST00000449934); *H. sapiens* MICB-001 (ENST00000252229); *Pteropus vampyrus* Putative MIC (ENSPVAT00000010513); *Mus musculus* Mill1-001 (ENSMUST00000066780); *M. musculus* Mill2-201 (ENSMUST00000072386) and *Rattus norvegicus* Mill1-201 (ENSRNOT00000035286).

## Additional Information

**How to cite this article**: Ng, J. H. J. *et al.* Evolution and comparative analysis of the bat MHC-I region. *Sci. Rep.* **6**, 21256; doi: 10.1038/srep21256 (2016).

## Supplementary Material

Supplementary Information

## Figures and Tables

**Table 1:** List of Annotated Genes in Bat MHC-I Region.

Description	Gene Symbol	Start	End	Strand	Accession	Locus Tag*
Olfactory Receptor 2H3	OR2H3	46088	47077	+	ELK11156	PAL_GLEAN10001315
Gamma-aminobutyric acid type B receptor subunit 1	GABBR1	80029	54804	—	ELK11157	PAL_GLEAN10001316
Myelin-oligodendrocyte glycoprotein	MOG	57164	111991	+	—	PAL_GLEAN10001317
NA	—	80999	80700	—	ELK11158	PAL_GLEAN10001318
DNA-directed RNA polymerase I subunit RPA12	ZNRD1	129247	133231	+	ELK11159	PAL_GLEAN10001320
Protein phosphatase 1 regulatory subunit 11	PPP1R11	133578	135422	+	ELK11160	PAL_GLEAN10001321
RING finger protein 39	RNF39	141652	137216	—	ELK11161	PAL_GLEAN10001322
Tripartite motif-containing protein 40	TRIM40	217223	225140	+	ELK11162	PAL_GLEAN10001323
Tripartite motif-containing protein 10	TRIM10	237670	231183	—	ELK11163	PAL_GLEAN10001324
Tripartite motif-containing protein 15	TRIM15	240064	247323	+	ELK11164	PAL_GLEAN10001325
Tripartite motif-containing protein 26	TRIM26	278910	260739	—	ELK11165	PAL_GLEAN10001326
Tripartite motif-containing protein 39	TRIM39	355894	365875	+	ELK11166	PAL_GLEAN10001327
Ribonuclease P protein subunit p21	RPP21	368494	370069	+	ELK11167	PAL_GLEAN10001328
60S ribosomal protein L17	RPL17	372779	398321	+	ELK11168	PAL_GLEAN10001329
Guanine nucleotide-binding protein-like 1	GNL1	407126	398634	—	ELK11169	PAL_GLEAN10001330
Proline-rich protein 3	PRR3	408251	412186	+	ELK11170	PAL_GLEAN10001331
ATP-binding cassette sub-family F member 1	ABCF1	421330	433377	+	ELK11171	PAL_GLEAN10001332
Serine/threonine-protein phosphatase 1 regulatory subunit 10	PPP1R10	447310	439370	—	ELK11172	PAL_GLEAN10001333
28S ribosomal protein S18b, mitochondrial	MRPS18B	454707	460056	+	ELK11173	PAL_GLEAN10001334
Alpha-tubulin N-acetyltransferase	ATAT1	460844	474884	+	ELK11174	PAL_GLEAN10001335
NA	—	475700	475921	+	ELK11175	PAL_GLEAN10001337
NA	—	477170	478927	+	ELK11176	PAL_GLEAN10001338
Putative pre-mRNA-splicing factor ATP-dependent RNA helicase DHX16	DHX16	493056	479259	—	ELK11177	PAL_GLEAN10001339
Phostensin	PPP1R18	502158	496328	—	ELK11178	PAL_GLEAN10001340
Nurim	NRM	507574	505324	—	ELK11179	PAL_GLEAN10001341
Mediator of DNA damage checkpoint protein 1	MDC1	520633	510638	—	ELK11180	PAL_GLEAN10001342
Tubulin beta-7 chain	TUBB2A	525446	528727	+	ELK11181	PAL_GLEAN10001343
Flotillin-1	FLOT1	540491	530782	—	ELK11182	PAL_GLEAN10001344
Radiation-inducible immediate-early gene IEX-1	IER3	542628	542066	—	ELK11183	PAL_GLEAN10001345
Epithelial discoidin domain-containing receptor 1	DDR1	656863	666873	+	ELK11184	PAL_GLEAN10001346
General transcription factor IIH subunit 4	GTF2H4	672626	677439	+	ELK11185	PAL_GLEAN10001347
Valyl-tRNA synthetase, mitochondrial	VARS2	678387	689535	+	ELK11186	PAL_GLEAN10001348
NA	—	690636	699283	+	ELK11187	PAL_GLEAN10001349
NA	—	709148	709528	+	ELK11188	PAL_GLEAN10001350
Diffuse panbronchiolitis critical region protein 1	DPCR1	710242	711886	+	ELK11189	PAL_GLEAN10001351
Mucin-21	MUC21	719741	741706	+	ELK11190	PAL_GLEAN10001353
Uncharacterised protein C6orf15	C6orf15	846751	845625	—	ELK11191	PAL_GLEAN10001356
Corneodesmosin	CDSN	853255	851625	—	ELK11192	PAL_GLEAN10001357
Psoriasis susceptibility 1 candidate gene 2 protein	PSORS1C2	869935	868930	—	ELK11193	PAL_GLEAN10001358
Coiled-coil alpha-helical rod protein 1	CCHCR1	894260	871986	—	ELK11194	PAL_GLEAN10001359
Transcription factor 19 (Partial)	TCF19	885867	927101	+	—	PAL_GLEAN10001360

^*^Locus tags refer to annotations in the *P. alecto* whole genome.

**Table 2:** List of Annotated BAC Clones.

BAC	Start	End	Strand	Description
Supercontig 1 P100M20 P103A18	26655	28273	+	Ubiquitin D (partial)
	54979	54038	—	LINE-1 Reverse Transcriptase like protein
	111183	113894	+	MHC Class I antigen Ptal-01*01
Supercontig 2 P212O7 P229M21	10838	13788	+	MHC Class I antigen *Ptal-05*
	41150	49360	+	Ubiquitin D (partial)
	80439	81369	+	Pro-pol like (*Bos taurus*)
	117948	120666	+	MHC Class I antigen Ptal-01*02
Supercontig 3 P201M3 P216K21	64954	63912	—	Elongation factor-1-α-1
	76996	78940	+	MHC Class I pseudogene *Ptal-06*^ψ^
	97999	101033	+	MHC Class I antigen *Ptal-04*
	126991	129739	+	Ubiquitin D
	133984	145987	+	Cortactin-binding protein 2 (partial)
	153839	152952	—	LINE-1 Reverse Transcriptase like protein
P56N20	18626	19514	+	Ubiquitin D
	90998	93706	+	MHC Class I antigen *Ptal-02*
	134350	137082	+	MHC Class I antigen *Ptal-03*
P56F16 Reference to MHC-I genomic region	77624	78187	+	Radiation-inducible immediate-early gene IEX-1
	79762	89472	+	Flotillin-1
	94809	91527	—	Tubulin beta-7 chain
	99622	109618	+	Mediator of DNA damage checkpoint protein 1
	112682	114933	+	Nurim
	118099	123930	+	Phostensin (PPP1R18)
	127202	141000	+	Putative pre-mRNA-splicing factor ATP-dependent RNA helicase DHX16

^ψ^ represents putative pseudogene.

**Table 3:** Coordinates of Bat Class I S-X-Y Motifs Within the BAC Clones.

Gene Name	C/SC*	Strand	Gene Start	S-X-Y Start	S-X-Y End	S-X-Y Relative Position (S-X-Y End to Gene Start)
Ptal-01*01	SC1	+	111183	111025	111088	−158
*Ptal-02*	P56N20	+	90998	90840	90903	−158
*Ptal-03*	P56N20	+	134350	134192	134255	−158
Ptal-01*02	SC2	+	117948	117781	117844	−167
*Ptal-05*	SC2	+	10838	10680	10743	−158
*Ptal-04*	SC3	+	97999	97848	97911	−151
*Ptal-06*^ψ^	SC3	+	76996	Not found	Not found	n/a

^ψ^ represents putative pseudogene.

^*^C represents contig while SC represents supercontigs.
