# Supplementary material for: Evolution and comparative analysis of the bat MHC-I region
Source: Sci Rep. 2016 Feb 15;6:21256. doi: 10.1038/srep21256 (PMC4753418; doi:10.1038/srep21256)
Supplement: Supplementary Information [file srep21256-s1.pdf]

Evolution and comparative analysis of the bat MHC-I region

Justin H. J. Ng[1,2,3]*, Mary Tachedjian[1], Janine Deakin[4], James W. Wynne[1], Jie Cui[3], Volker Haring[5], Ivano Broz[5], Honglei Chen[5], Katherine Belov[2], Lin-Fa Wang[3] and Michelle L. Baker[1]*

1. CSIRO Health and Biosecurity Business Unit, Australian Animal Health Laboratory, Geelong, VIC 3220, Australia

2. Faculty of Veterinary Science, University of Sydney, NSW 2006, Australia

3. Program in Emerging Infectious Diseases, Duke-National University of Singapore Medical School, Singapore 169857

4. Institute for Applied Ecology, The University of Canberra, ACT 2617, Australia

5. CSIRO, Australian Animal Health Laboratory, Geelong, VIC 3220, Australia

***Corresponding authors**

Correspondence to:

Michelle L. Baker

CSIRO Australian Animal Health Laboratory,

Geelong, Victoria 3220, Australia

P: +61 03 5227 5052

F: +61 03 5227 5555

E: michelle.baker@csiro.au

Justin H. J. Ng

Program in Emerging Infectious Diseases,

Duke-National University of Singapore Medical School,

Singapore 169857

P: +65 9002 5606

F: +65 6221 8147

E: justin.ng@duke-nus.edu.sg

Supplementary Figure 1. Dotplot analysis of BAC contig and supercontigs.

(a) Supercontig 1 vs supercontig 2, (b) supercontig 1 vs supercontig 3, (c) supercontig 2 vs supercontig 3, (d) supercontig 1 vs contig P56N20, (e) supercontig 2 vs contig P56N20, (f) supercontig 3 vs contig P56N20.

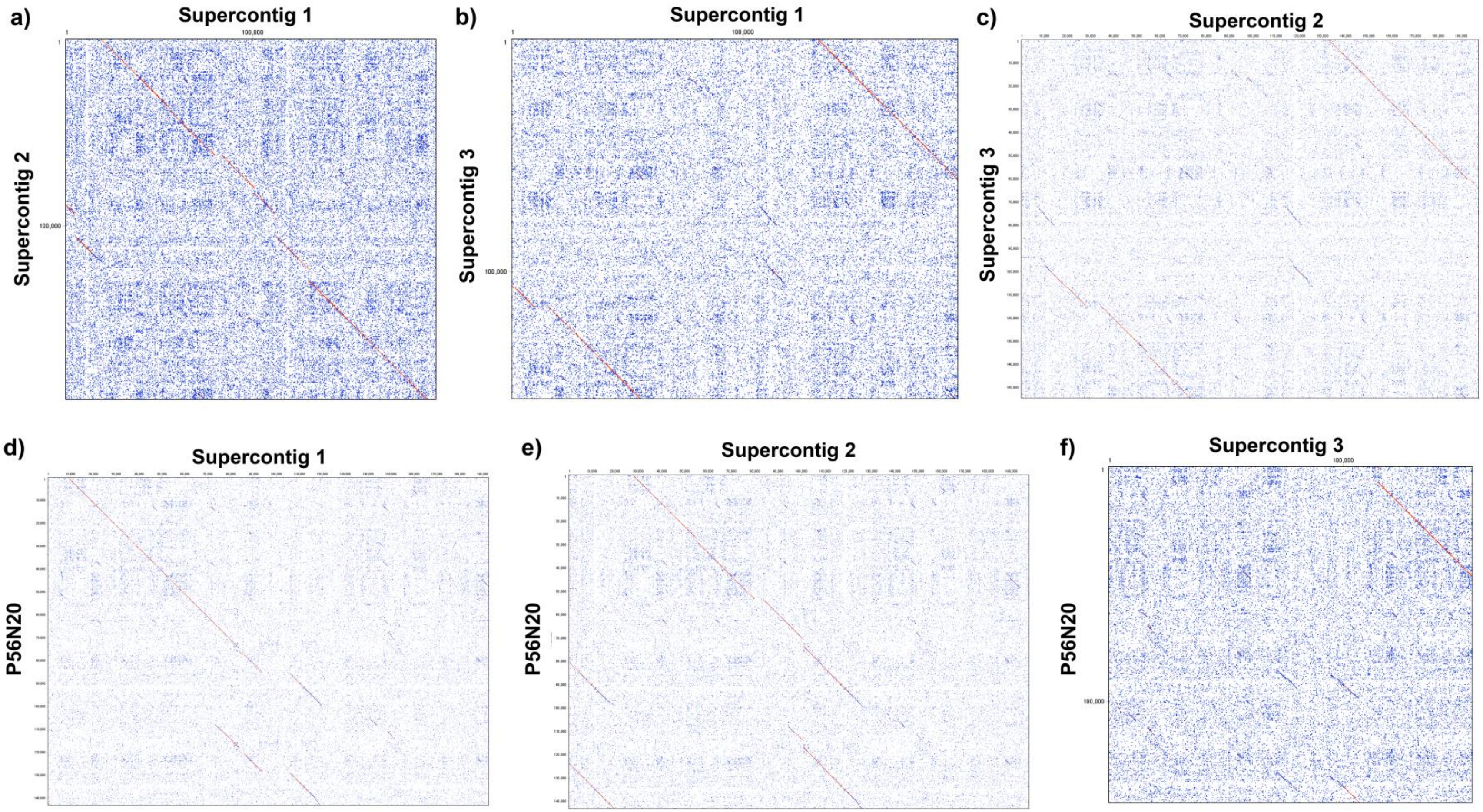
a)
Supercontig 1
Supercontig 2
100,000
b)
Supercontig 1
Supercontig 3
100,000
c)
Supercontig 2
Supercontig 3
d)
Supercontig 1
P56N20
e)
Supercontig 2
P56N20
f)
Supercontig 3
P56N20
100,000

Supplementary Figure 2: Alignment of deduced amino acid of bat MHC-I sequences with HLA genes.

Dashes indicate identical residues; Dots indicate gaps; P Peptide-Binding Sites; B β2-microglobulin Interaction Sites; 8 CD8 Interaction Sites; C Cysteine residues in α1 and α2 domains likely to form intra-chain disulphide bonds; NK Putative natural killer (NK) receptor-binding region; GGG glycosylation site. ψ represents putative pseudogene.

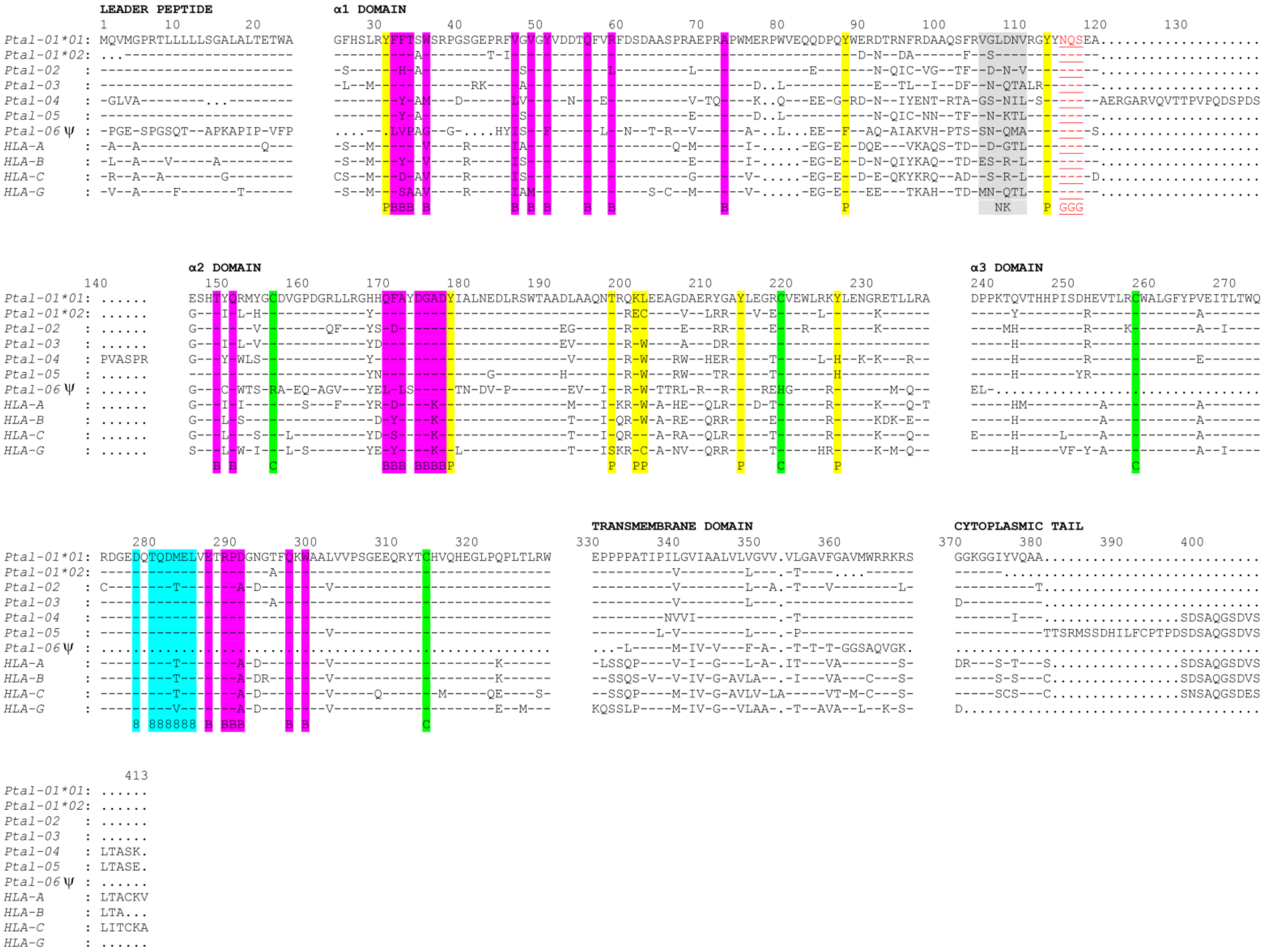
LEADER PEPTIDE
α1 DOMAIN
α2 DOMAIN
α3 DOMAIN
TRANSMEMBRANE DOMAIN
CYTOPLASMIC TAIL
Ptal-01*01
Ptal-01*02
Ptal-02
Ptal-03
Ptal-04
Ptal-05
Ptal-06 ψ
HLA-A
HLA-B
HLA-C
HLA-G

Supplementary Figure 3. Analysis of MHC-I genes and transcripts (α1 domain).

(a) Maximum likelihood phylogeny was used based on alignment of nucleotide sequences to exon 2, corresponding to α1 domain. HKY with discrete Gamma distribution was used to model evolutionary rate differences among sites (5 categories (+*G*, parameter = 1.5470)). The tree is drawn to scale, with branch lengths representing the number of substitutions per site. Branch support is indicated as percentage of trees out of 1000 bootstrap replicates that produce the same branching order. *Ptal – Pteropus alecto*. ψ represents putative pseudogene.

(b) Alignment of deduced amino acid of bat MHC-I genes and transcripts.

Sequences boxed in red represent region where amino acid insertions are locate.

Dashes indicate identical residues; Dots indicate gaps; P Peptide-Binding Sites; GGG glycosylation site and ψ represents putative pseudogene.

a)

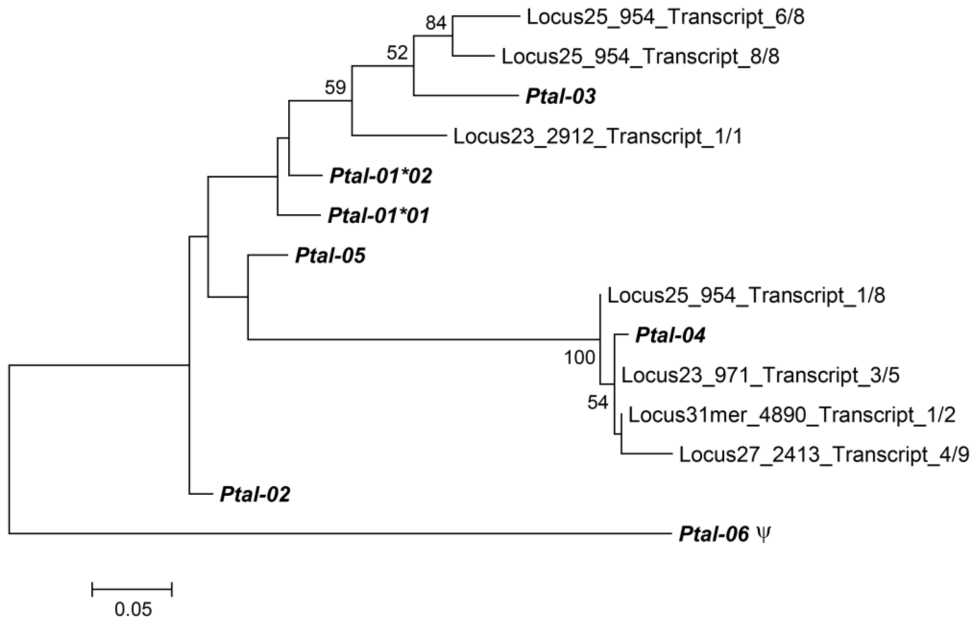

b)

```
                                 1        10        20        30        40        50        60        70        80        90   95
Ptal-01*01                     : GFHSLRYFFTSWSRPGSGEPRFVGVGYVDDTQFVRFDSDAASPRAEPRAPWMERPWVEQQDPQYWERDTRNFRDAAQSFRVGLDNVRGYYNQSEA
Ptal-01*02                     : ----------A--------T-I-------------------------------------------D-N--DA---------S-------------
Ptal-02                        : -S------H-A------------S----------L---------L--------------E-------N-QIC-VG--T---D-N-----------
Ptal-03                        : -L--M------------RK----A----------------------------D..L-----------E--TL--I--D---N-QTALR-------
Ptal-04                        : --------Y-AM---D------LV-----N---E----------V-TQ----K..Q---EE-G-RD-N--IYENT-RTA-GS-NIL-S-------
Ptal-05                        : --------Y-A------------S--------------------E-------D..L-----------N-QIC-NN--T---N-KTL---------
Ptal-06 Ψ                      : KAPIPEV-PLRLVPA--RGGHYIS--F------L--N--T-R--V------A-.....LEE--F--AQ-AIAKVH-PTS-SN-QMA--------S
Locus23_971_Transcript_3/5     : --------Y-AM---D------LV---------E----------V-TQ----K..Q---EE-G-RD-N--IYENT-RTA-GS-NIL-S-------
Locus31mer_4890_Transcript_1/2 : --------Y-AM---D------LV---------E----------V-TQ----K..Q---EE-G-RD-N--IYENT-RTA-GS-NIL-S-------
Locus25_954_Transcript_1/8     : ..........XM---D------LV--------------------V-TQ----K..Q---EE-G-RD-N--IYENT-RTA-GS-NIL-S-------
Locus25_954_Transcript_6/8     : .------YY-A--Q-----T--IS-S-------S-----N------------D..L-----------N---A-----TY--N-QTALR-------
Locus25_954_Transcript_8/8     : ..........XM---D------LV---------------N------------D..L-----------N---A-----TY--N-QTALR-------
Locus23_2912_Transcript_1/1    : ----M------------------A---------L-----N------------D..L--------RD-H---A--I--D------D-LR-------
Locus27_2413_Transcript_4/9    : .............ARPR-----LV---------E-------T--V-TQ----K..Q---EE-G-RD-N--IYENT-RTA-GS-NIL-S-------
                                       P                                                        P                        P GGG
```

Supplementary Figure 4. Phylogenetic tree of MHC-I genes.

a) Hyper-variable domain (α1 and α2 domains).

Maximum likelihood phylogeny was used based on alignment of nucleotide sequences to exons 2 and 3, corresponding to α1 and α2 domains respectively. HKY with discrete Gamma distribution was used to model evolutionary rate differences among sites (5 categories (+*G*, parameter = 1.5470)). The tree is drawn to scale, with branch lengths representing the number of substitutions per site. Branch support is indicated as percentage of trees out of 1000 bootstrap replicates that produce the same branching order.

b) Constant domain (α3 domain).

Maximum likelihood phylogeny was used based on alignment of nucleotide sequences to exon 4, corresponding to α3 domain. HKY with discrete Gamma distribution was used to model evolutionary rate differences among sites (5 categories (+*G*, parameter = 1.5470)). The tree is drawn to scale, with branch lengths representing the number of substitutions per site. Branch support is indicated as percentage of trees out of 1000 bootstrap replicates that produce the same branching order.

c) MHC-I α chain (α1 – α3 domains) using Neighbour-Joining.

Neighbour-Joining phylogeny was used based on alignment of nucleotide sequences to exons 2, 3 and 4, corresponding to α1, α2 and α3 domains respectively. Maximum Composite Likelihood with discrete Gamma distribution was used to model evolutionary rate differences among sites (+*G*, parameter = 1). The tree is drawn to scale, with branch lengths representing the number of substitutions per site. Branch support is indicated as percentage of trees out of 1000 bootstrap replicates that produce the same branching order.

d) MHC-I α chain (α1 – α3 domains) using Minimum Evolution.

Minimum Evolution phylogeny was used based on alignment of nucleotide sequences to exons 2, 3 and 4, corresponding to α1, α2 and α3 domains respectively. Maximum Composite Likelihood with discrete Gamma distribution was used to model evolutionary rate differences among sites (+*G*, parameter = 1). The tree is drawn to scale, with branch lengths representing the number of substitutions per site. Branch support is indicated as percentage of trees out of 1000 bootstrap replicates that produce the same branching order.

SLA – swine leucocyte antigen; *EQMHC* – equine MHC; *DLA* – dog leucocyte antigen; *HLA* – human leucocyte antigen; *Bota – Bos taurus*; *Modo – Monodelphis domestica*; *Oran – Ornithorhynchus anatinus*; *Gaga – Gallus gallus*; *Hosa – Homo sapiens*; *Ptal – Pteropus alecto*; *Ptva – Pteropus vampyrus*; *Mumu – Mus musculus*; *Rano – Rattus norvegicus*.

The non-classical MHC-like, human *MIC* and rodent *Mill*, genes were used as outgroups. A putative *MIC* ortholog from the *P. vampyrus* genome was also included in the analysis.

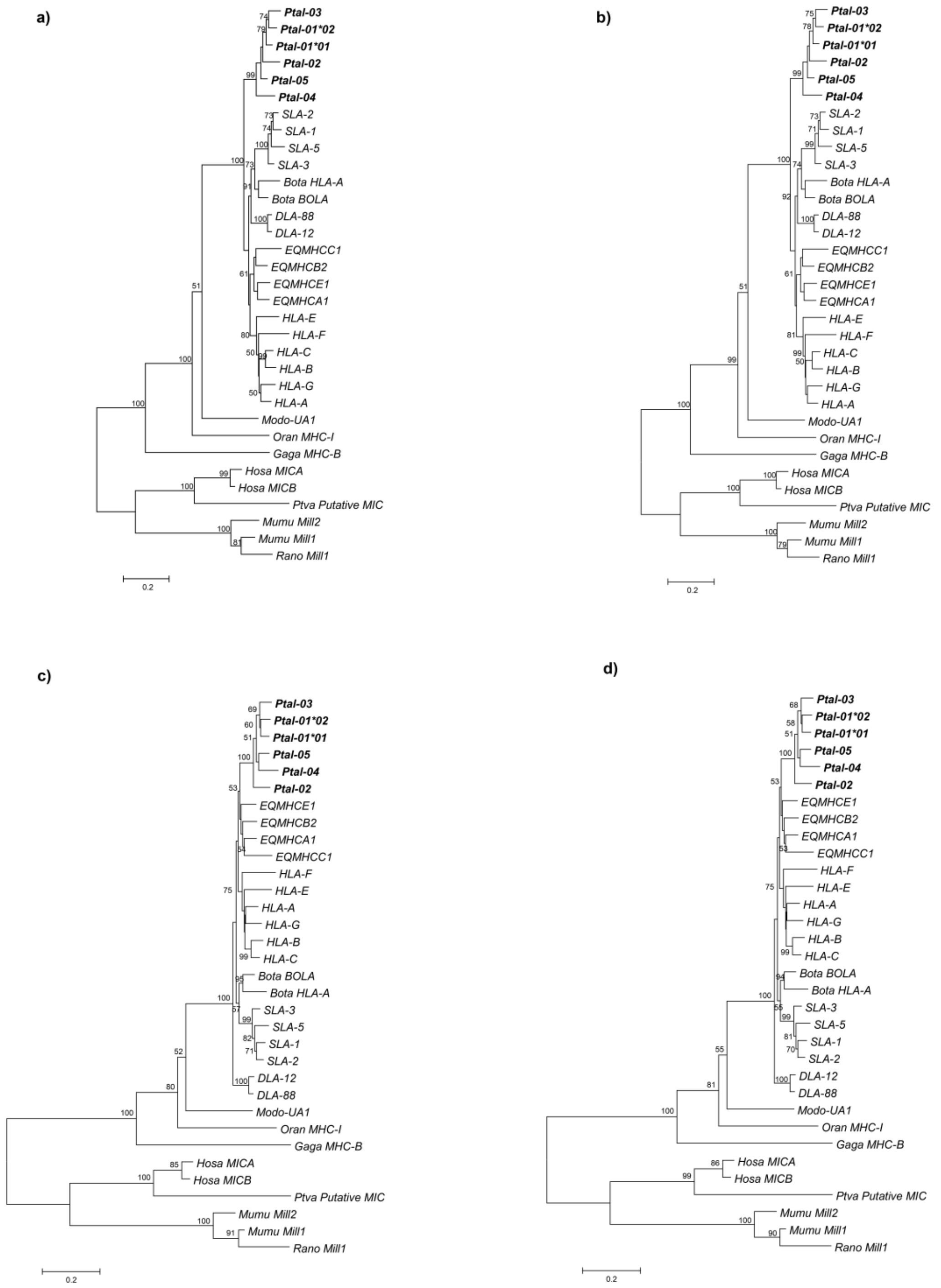

a)
Ptal-03
Ptal-01*02
Ptal-01*01
Ptal-02
Ptal-05
Ptal-04
SLA-2
SLA-1
SLA-5
SLA-3
Bota HLA-A
Bota BOLA
DLA-88
DLA-12
EQMHCC1
EQMHCB2
EQMHCE1
EQMHCA1
HLA-E
HLA-F
HLA-C
HLA-B
HLA-G
HLA-A
Modo-UA1
Oran MHC-I
Gaga MHC-B
Hosa MICA
Hosa MICB
Ptva Putative MIC
Mumu Mill2
Mumu Mill1
Rano Mill1
0.2
b)
Ptal-03
Ptal-01*02
Ptal-01*01
Ptal-02
Ptal-05
Ptal-04
SLA-2
SLA-1
SLA-5
SLA-3
Bota HLA-A
Bota BOLA
DLA-88
DLA-12
EQMHCC1
EQMHCB2
EQMHCE1
EQMHCA1
HLA-E
HLA-F
HLA-C
HLA-B
HLA-G
HLA-A
Modo-UA1
Oran MHC-I
Gaga MHC-B
Hosa MICA
Hosa MICB
Ptva Putative MIC
Mumu Mill2
Mumu Mill1
Rano Mill1
0.2
c)
Ptal-03
Ptal-01*02
Ptal-01*01
Ptal-05
Ptal-04
Ptal-02
EQMHCE1
EQMHCB2
EQMHCA1
EQMHCC1
HLA-F
HLA-E
HLA-A
HLA-G
HLA-B
HLA-C
Bota BOLA
Bota HLA-A
SLA-3
SLA-5
SLA-1
SLA-2
DLA-12
DLA-88
Modo-UA1
Oran MHC-I
Gaga MHC-B
Hosa MICA
Hosa MICB
Ptva Putative MIC
Mumu Mill2
Mumu Mill1
Rano Mill1
0.2
d)
Ptal-03
Ptal-01*02
Ptal-01*01
Ptal-05
Ptal-04
Ptal-02
EQMHCE1
EQMHCB2
EQMHCA1
EQMHCC1
HLA-F
HLA-E
HLA-A
HLA-G
HLA-B
HLA-C
Bota BOLA
Bota HLA-A
SLA-3
SLA-5
SLA-1
SLA-2
DLA-12
DLA-88
Modo-UA1
Oran MHC-I
Gaga MHC-B
Hosa MICA
Hosa MICB
Ptva Putative MIC
Mumu Mill2
Mumu Mill1
Rano Mill1
0.2

Supplementary Figure 5. Predicted protein models of representative bat MHC-I genes.

(a) *Ptal-01* and (b) *Ptal-03*.

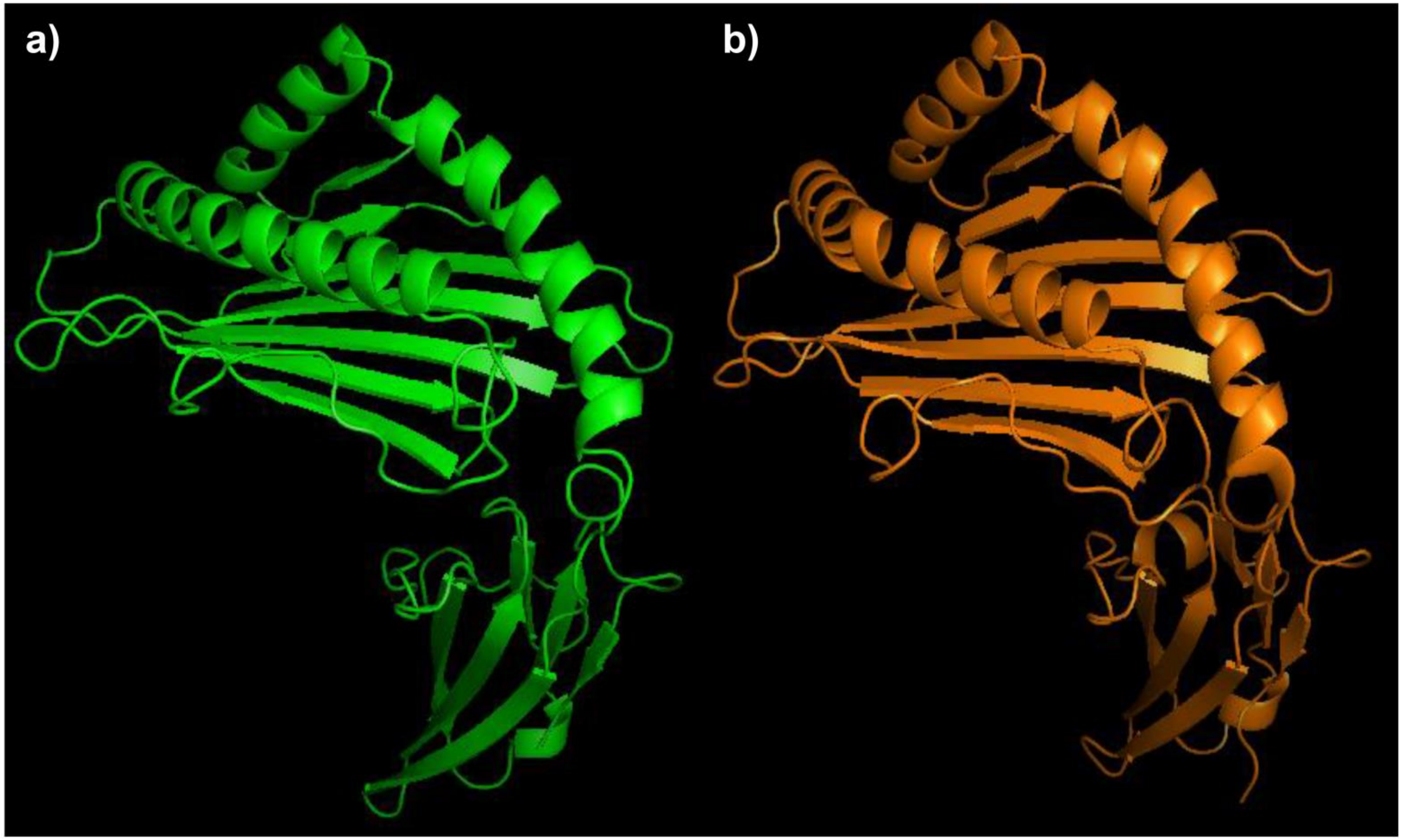

Supplementary Table 1: Overgo Sequences for BAC Probing.

Overgo	5' → 3' Sequence
MHC-I α3 Consensus - OVa	CAA AGA CAC ACG TGA CCC ACC ACC
MHC-I α3 Consensus - OVb	TCA TGG TCA GAG ATG GGG TGG TGG
FLOT1 - OVa	TTT CAC CTG TGG TCC AAA TGA GGC
FLOT1 - OVb	ACC GGA GAC CAC CAT GGC CTC ATT
GTF2H4 - OVa	TCC GTA TCT GGC ACA CTC AGC TGC
GTF2H4 - OVb	TGG AGA CCA CCA GGG AGC AGC TGA
MICA - OVa	TCC AAG TCC TGC TGC TTG CTG GTG
MICA - OVb	GAT CCA AAA AGG GCA GCA CCA GCA
POU5F1B - OVa	ATG GCG GGA CAC CTG GCT TCC GAC
POU5F1B - OVb	GGG GCG AAA AGG CAA AGT CGG AAG
TRIM10 - OVa	TCT GTG ACC AGC CTG GCA GAT GAG
TRIM10 - OVb	AGA TGG GGC AGT TGA CCT CAT CTG
TRIM26 - OVa	ACA TCA GCA CCA CTG CGG AGC CTG
TRIM26 - OVb	AGG TCA CCT CCT CCT CCA GGC TCC
ABCF1 - OVa	GCT AGA GAA GTT CAG CAT CTC AGC
ABCF1 - OVb	TAA CTC CTT GCC ATG GGC TGA GAT
PA-TCF19 - OVa	GTC ACA GGC TGG AGT TGA GTG ATG
PA-TCF19 - OVb	AAG GTC AGA AGG TCA CCA TCA CTC
PA-MOG - OVa	GGT GGT TCA TCT CTA CCG AAA TGG
PA-MOG - OVb	TCC ATC CTG ATC CTT GCC ATT TCG
PA-GABBR1 - OVa	GTT AAA GGA CAG ACA GAC TAC AGC
PA-GABBR1 - OVb	CCT GGG TAT TGT CCT AGC TGT AGT
PA-DCPR1 - OVa	TGG GCC ATT GTG TTG ATC ACT CTC
PA-DCPR1 - OVb	TTG CTG CCA CCA AAG TGA GAG TGA
PA-CDSN - OVa	CAC TCA ACG TTG AGG AAG ACA CAG
PA-CDSN - OVb	GGT CAC CCT TGC ATT TCT GTG TCT

Supplementary Table 2: BAC Primer Sequences for PCR Amplification.

Primer	5' ⟶ 3' Sequence
α3 (Consensus)-F	TGA GGT CAC CCT GAG GTG C
α3 (Consensus)-R	GGG CAG CCC CTC ATG CTG
ABCF1-F	GAA AGA GCT CAT GGA GCG TC
ABCF1-R	TCT TTC TCT TCA TTA TCC ATA GC
FLOT1-F	CAT GGG GTC CCC ATC TCA G
FLOT1-R	GAG TGC AAA TAG TCC TG
GTF2H4-F	ACA GGG CTG CTG AGC AGC
GTF2H4-R	CAA CAT AAA GTA CCA GAG CTG
MICA-R	TTC CAG AGG GCA CAG GGT G
MICA-F	AGT CTT CGT TAT AAC CTC ATG G
PA-CDSN-F	CCA GGA TGT CCC GGA TGG
PA-CDSN-R	CAT CGT GTT CCA GCC AGT G
PA-DPCR1-F	GGG GTG GAT TTG AGG TCC
PA-DCPR1-R	GCG TGC TGG TCA GTC TCC
PA-GABBR1-F	GAG ACA CCC TCA AAG GAC G
PA-GABBR1-R	AGA CAC CCT GAG GAG ACA G
PA-MOG-F	ACA GTT CAG AGT AAT AGG ACC
PA-MOG-R	ACT TTC AAT TCC ATT GCT GCC
PA-TCF19-F	TGG TCA ATA ATG TTC GAC TCC
PA-TCF19-R	CCA CGA GGT GTC AGT GGG
POU5F1B-F	CGC TTT GAG GCT CTG CAG C
POU5F1B-R	GGG CGA TGT GGC TGA TCT G
PPP1R1-F	GAG ACA ACG GTT ACC GTG AC
PPP1R11-R	TGG GGT TGC ACG ACT CCG
TRIM10-F	GAT GAG ATG CAG TTG TGC GT
TRIM26-F	CAG AGC TCA TGC AGG ACA C
TRIM10-R	TGG GCT AAG AGG ATG CTC TG
TRIM26-R	AGT CCT CTG ACA GCT GC AG
TUBB-F	ACC CAA AGT GTC TGA CAC CG
TUBB-R	AAG GGC ACC ATG TTG ACT GC

Supplementary Table 3: 454 FLX+ Output Data Statistics.

BAC Clone	Average Read Length (bp)	# Reads (Raw)	# Reads (Clean)
P56F16	673	87,058	82,227
P56N20	711	78,046	73,926
P100M20	708	62,220	59,666
P103A18	702	87,226	82,392
P201M3	673	168,727	157,824
P212O7	701	172,259	165,207
P216K21	700	127,257	118,209
P229M21	693	167,287	157,139

Supplementary Table 4: Comparison of Nucleotides (green) and Amino Acid (blue) Residues of α1 – α3 Domains of Bat MHC-I Genes.

NT \ AA	*Ptal-01*01*	*Ptal-01*02*	*Ptal-02*	*Ptal-03*	*Ptal-04*	*Ptal-05*
Ptal-01*01	100%	89.96%	84.59%	87.73%	80.14%	88.09%
Ptal-01*02	94.74%	100%	84.59%	86.64%	81.59%	85.20%
Ptal-02	90.92%	90.68%	100%	82.67%	78.34%	85.92%
Ptal-03	93.62%	93.26%	88.93%	100%	79.42%	87.36%
Ptal-04	89.53%	89.29%	86.28%	88.45%	100%	84.12%
Ptal-05	94.10%	92.78%	91.10%	92.90%	91.34%	100%

Supplementary Table 5: Comparison of Nucleotides (green) and Amino Acid (blue) Residues of α1 – α2 Domains of Bat MHC-I Genes.

NT \ AA	*Ptal-01*01*	*Ptal-01*02*	*Ptal-02*	*Ptal-03*	*Ptal-04*	*Ptal-05*	*Ptal-06*
Ptal-01*01	100%	87.17%	82.89%	83.24%	71.89%	84.32%	54.91%
Ptal-01*02	93.23%	100%	82.35%	81.08%	74.05%	80.54%	54.91%
Ptal-02	91.09%	90.73%	100%	79.46%	72.43%	83.78%	57.80%
Ptal-03	90.99%	90.45%	87.93%	100%	70.27%	82.70%	56.40%
Ptal-04	85.05%	84.86%	83.60%	83.24%	100%	77.84%	54.07%
Ptal-05	92.07%	90.45%	90.81%	90.09%	87.57%	100%	58.72%
Ptal-06	70.71%	70.91%	71.10%	71.71%	69.96%	71.51%	100%

Supplementary Table 6: Root Mean Square Deviation (RMSD) of Various Resolved and Predicted MHC-I Molecule Models.

Gene	Resolution (Å)	PDB ID	RMSD				
			3LN4	*3BXN*	*2XPG*	*3BVN*	*3JTS*
HLA-B	1.30	*3LN4*	0	-	-	-	-
HLA-B	2.50	*1MI5*	0.751	-	-	-	-
HLA-B	2.55	*3BVN*	0.597	-	-	0	-
HLA-B	1.86	*3BXN*	0.215	0	-	-	-
HLA-A	2.60	*2XPG*	0.532	-	0	-	-
Mamu MHC-I	2.80	*3JTS*	0.605	-	-	-	0
Mumu H2-K	2.60	*2FWO*	0.650	-	-	-	-
Bota MHC-I	1.90	*2XFX*	0.821	-	-	-	-
SLA-1	2.10	*3QQ4*	0.637	-	-	-	-
Gaga MHC-I	2.90	*2YEZ*	2.334	-	-	-	-
Eqca MHCA1	Predicted	**3BXN*	0.514	0.460	-	-	-
DLA-12	Predicted	**2XPG*	0.724	-	0.502	-	-
DLA-64	Predicted	**3BVN*	0.768	-	-	0.542	-
Modo-02	Predicted	**3LN4*	0.514	-	-	-	-
Ptal-01	Predicted	**3LN4*	0.373	-	-	-	-
Ptal-03	Predicted	**3JTS*	0.786	-	-	-	0.538

Mamu – *Macaca mulatta*; Mumu – *Mus musculus*; Bota – *Bos taurus*; Gaga – *Gallus gallus*; Eqca – *Equus caballus*.
*PDB ID used as query for predicted protein models.
